# Recapitulation of physiologic and pathophysiologic pulsatile CSF flow in purpose-built high-throughput hydrocephalus bioreactors

**DOI:** 10.1186/s12987-024-00600-1

**Published:** 2024-12-19

**Authors:** Ahmad Faryami, Adam Menkara, Shaheer Ajaz, Christopher Roberts, Ryan Jaroudi, Blake Gura, Tala Hussini, Carolyn A. Harris

**Affiliations:** 1 Department of Chemical Engineering and Materials Science, Wayne State University, 6135 Woodward Avenue, Rm 1413, Detroit, MI 48202 USA; 2 Department of Biomedical Engineering, Wayne State University, 818 W Hancock St, Detroit, MI 48201 USA; 3 Department of Biomedical Engineering, University of Michigan, 2200 Bonisteel Blvd, Ann Arbor, MI 48109 USA

**Keywords:** Hydrocephalus, In vitro modeling, Cerebrospinal fluid flow pattern, CSF dynamics, Microfluidic, Pump, CSF amplitude, Bioreactor chamber, Benchtop model.

## Abstract

**Background:**

Hydrocephalus, an accumulation of cerebrospinal fluid (CSF) in the ventricles of the brain, is often treated via a shunt system to divert the excess CSF to a different compartment; if left untreated, it can lead to serious complications and permanent brain damage. It is estimated that one in every 500 people are born with hydrocephalus. Despite more than 60 years of concerted efforts, shunts still have the highest failure rate of any neurological device requiring follow-up shunt revision surgeries and contributing to the $2 billion cost of hydrocephalus care in the US alone. The absence of a tested and validated long-term in-vitro model that can incorporate clinically relevant parameters has limited hypothesis-driven studies and, in turn, limited our progress in understanding the mechanisms of shunt obstruction in hydrocephalus. Testing clinical parameters of flow, pressure, shear, catheter material, surface modifications, and others while optimizing for minimal protein, cellular, and blood interactions has yet to be done systematically for ventricular catheters. Several studies point to the need to not only understand how cells and tissues have occluded these shunt catheters but also how to stop the likely multi-faceted failure. For instance, studies show us that tissue occluding the ventricular catheter is primarily composed of proliferating astrocytes and cells of the macrophage lineage. Cell reactivity has been observed to follow flow gradients, with elevated levels of typically pro-inflammatory interleukin-6 produced under shear stress conditions greater than 0.5 dyne/$$\:{cm}^{2}$$. But also, that shear can shift cellular attachment. The *A*utomated, *I*n vitro *M*odel for hydrocephalu*s* research (AIMS), presented here, improves upon our previous long-term in vitro systems with specific goals of recapitulating bulk pulsatile cerebrospinal fluid (CSF) waveforms and steady-state flow directionality relevant to ventricular catheters used in hydrocephalus.

**Methods:**

The AIMS setup was developed to recapitulate a wide range of physiologic and pathophysiologic CSF flow patterns with varying pulse amplitude, pulsation rate, and bulk flow rate with high throughput capabilities. These variables were specified in a custom-built user interface to match clinical CSF flow measurements. In addition to flow simulation capabilities, AIMS was developed as a modular setup for chamber testing and quality control. In this study, the capacity and consistency of single inlet resin chambers (*N* = 40), multidirectional resin chambers (*N* = 5), silicone chambers (*N* = 40), and PETG chambers (*N* = 50) were investigated. The impact of the internal geometry of the chamber types on flow vectors during pulsatile physiologic and pathophysiologic flow was visualized using Computational Fluid Dynamics (CFD). Dynamic changes in ventricular volume were investigated by combining AIMS with MRI-driven silicone model of a pediatric patient’s ventricles. Parametric data were analyzed using one-way analysis of variance (ANOVA) or repeated measures ANOVA tests. Non-parametric data were analyzed using Kruskal-Wallis test. For all tests, a confidence interval was set at 0.95 (α = 0.05). In a subset of experiments, AIMS was also tested for its capability to measure the flow of florescent microspheres through the holes of unused and explanted ventricular catheters.

**Results:**

The analysis of peak amplitude through chambers indicated no statistically significant differences between the chamber batches. This high throughput setup was able to reproduce clinical measurements of bulk CSF flow tested in up to 50 independent pump channels such that there was no exchange of solution or flow interference between adjacent channels. Physiologic and pathophysiologic clinical measurements of CSF flow patterns were recapitulated in all four chamber types of the AIMS setup with and without augmented compliance. The AIMS setup’s automated priming feature facilitated constant fluid contact throughout the study; no leaks or ruptures were observed during short- (up to 24 h) or long-term (30 days) experiments. Finally, qualitative microscopy long-exposure image capture revealed microsphere movement under steady-state and pulsatile flow of spheres moving into the shunt catheter.

**Conclusion:**

AIMS successfully simulates clinical measurements of physiologic and pathophysiologic CSF pulsation amplitude and frequency, as exemplified using clinical data of CSF exiting an externalized ventricular drain in four distinct chamber types, as well as flow patterns from a valve. This provides a promising platform for investigating the direct interaction between CSF, immune cells, and shunt hardware under relevant flow conditions when both the source of bulk flow and pulsatility are coupled. The implementation of this system in conjunction with a previously reported three-dimensional hydrogel scaffold in future work will enhance our understanding of shunt-related complications and improve treatment strategies by reducing the obstruction rate.

**Supplementary Information:**

The online version contains supplementary material available at 10.1186/s12987-024-00600-1.

## Background

This work presents the development and testing of the fundamental tools necessary for the investigation of shunt failure in vitro under relevant flow conditions. Hydrocephalus, an accumulation of cerebrospinal fluid (CSF) in the ventricles of the brain, is a treatable neurological condition; if left untreated, it can lead to serious complications and permanent brain damage. It is estimated that one in every 500 people are born with hydrocephalus [1]. Acquired hydrocephalus after birth, including normal pressure hydrocephalus occurring significantly after birth, can present with some similar clinical symptoms. Most hydrocephalus patients are treated with the surgical placement of a shunt system to drain excess CSF. Unfortunately, the shunt system often fails due to obstruction of the outflow pathway of the ventricular catheter. Despite more than 60 years of concerted efforts at improving the longevity of ventricular catheters, shunts still have the highest failure rate of any neurological device: 50% of all shunts fail after two years, which rises to 85% after ten years [2, 3]. Although obstruction is the leading cause of failure, shunts are also susceptible to other complications such as infection, migration, and disconnection requiring follow-up shunt revision surgeries and contributing to the $2 billion cost of hydrocephalus care in the US alone [4–7].

A variety of methods including in vitro experiments, in vivo animal studies, translational work, and clinical studies have been utilized to identify the mechanism of obstruction formation that preludes shunt failure and subsequent revision. Many of these studies have primarily focused on endpoint analysis of the catheter without incorporating dynamic, quantitative, or qualitative outcomes throughout the study or the life of the catheter [8–13]. Ethical considerations, small sample size, and high cost are some of the limitations of animal models for hydrocephalus research despite their obvious clinical relevance. The simplicity of in vitro assays allows for increased sample size and the examination of specific cause-effect mechanistic relationships, but accurate replication of the complex in-vivo environment has proven to be a significant limitation [14–17]. The absence of a comprehensive, long-term model that incorporates many of the environmental constraints is currently a significant barrier to progress in understanding the pathogenesis of shunt failure and improving treatment.

Previous studies have proposed bioreactors as an intermediary between computer models and complex biological systems in the context of hydrocephalus treatment [18, 19]. The work presented here is the development and testing of the *A*utomated, *I*n vitro *M*odel for hydrocephalu*s* research (AIMS). AIMS is designed as a modular, high-throughput testing platform for recapitulating patient-specific in vivo hydrocephalus conditions and rigorous real-time data collection across as many as 50 concurrent samples.

Furthermore, dynamic changes in ventricular volume were investigated by combining AIMS with MRI-driven silicone model of a pediatric patient’s ventricles. This investigation marked the initial step in the development, testing and validation of an in-vitro hydrocephalus model with an overall goal to obtain insights into the interaction between the shunt systems, CSF dynamics and hydrocephalus pathophysiology leading to underdrainage and overdrainage.

## Materials and methods

### Bioreactor chambers manufacturing and processing

**Fig. 1:**
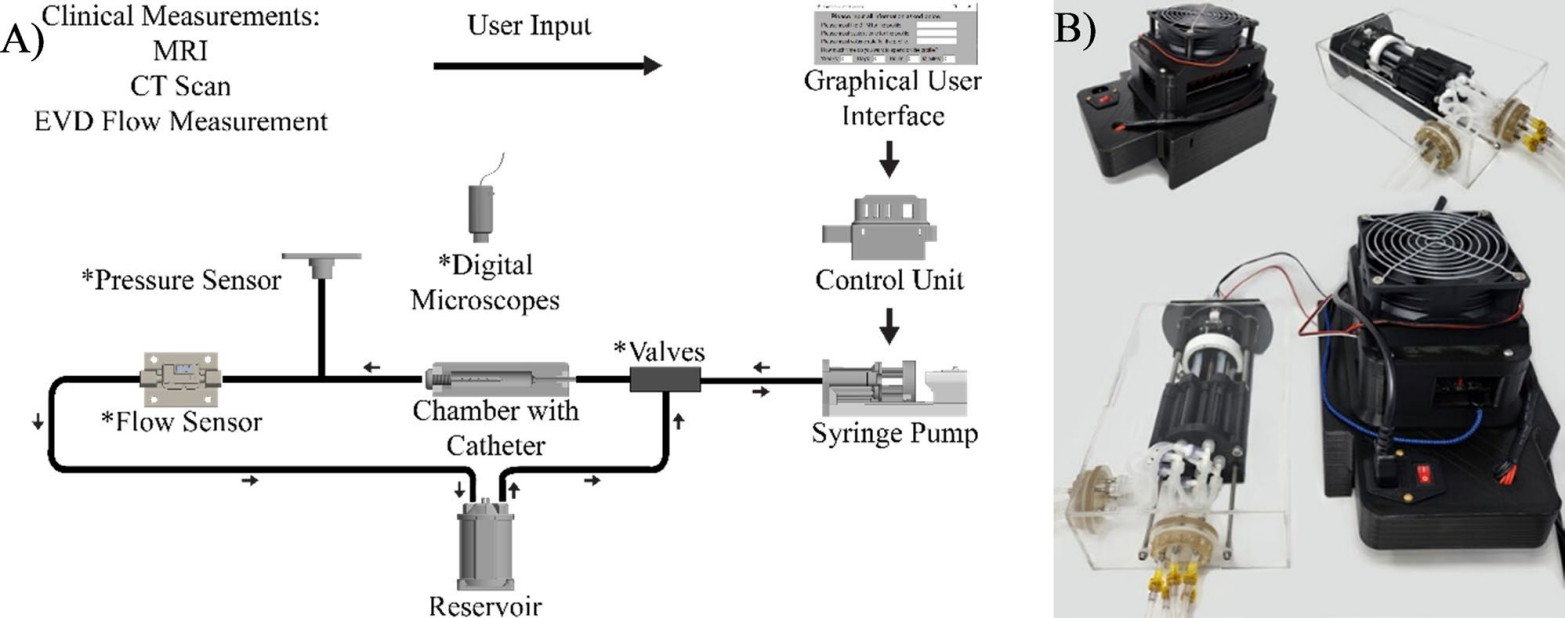
The overview diagram. (**A**) Schematic diagram illustrating the flow of information and fluids; from user input to measurable outcomes. (**B**) A fully assembled Control Unit and the Reciprocating Positive Displacement Pumps. Chamber with catheter not shown

AIMS was composed of three main components: (1) interchangeable bioreactor chambers, (2) a control unit, and (3) a custom-built positive displacement pump (Fig. 1, S. Figure 1) [17, 20].

**Fig. 2:**
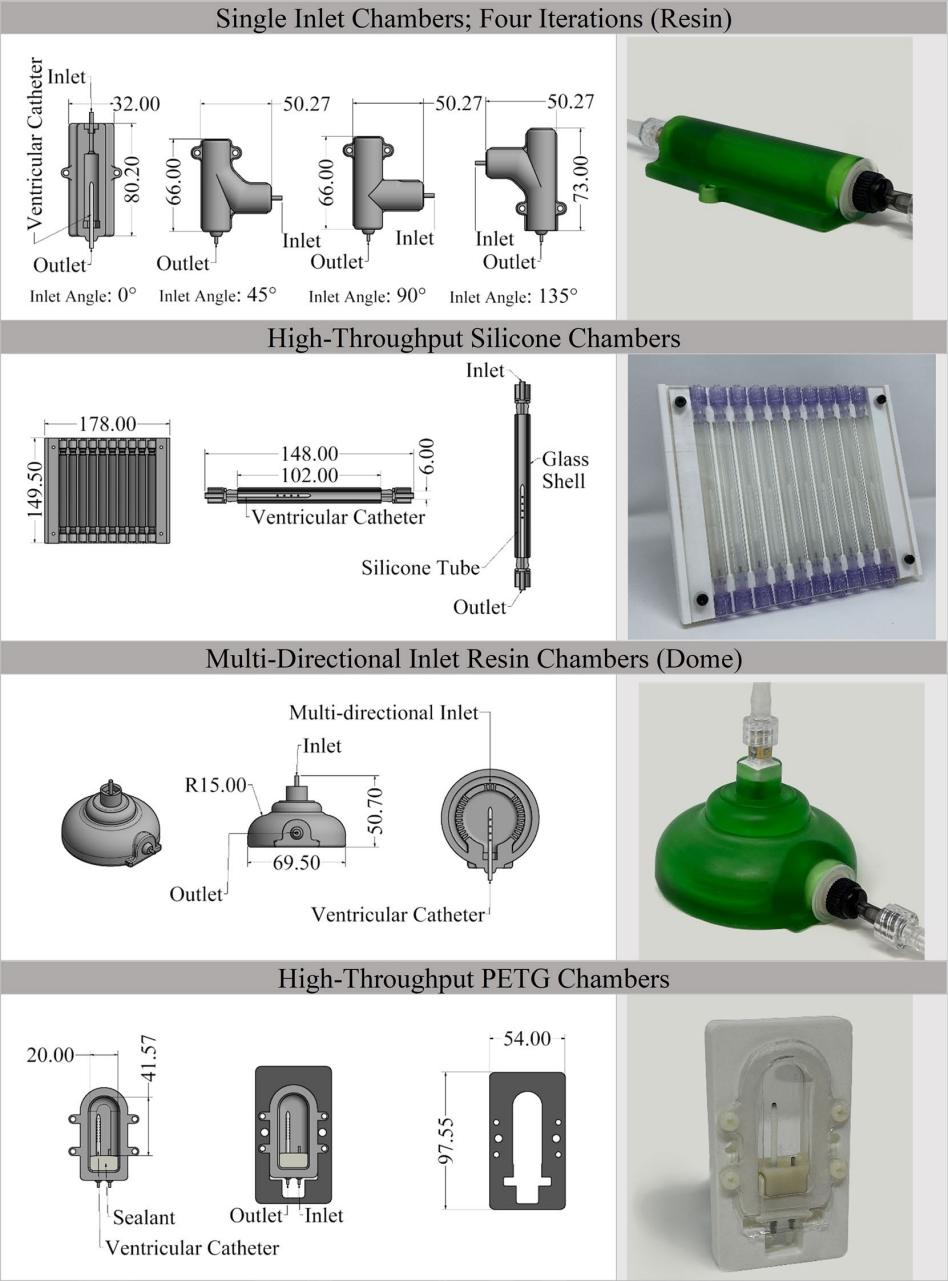
Schematic of the bioreactor chambers. Dimensions and specifications of bioreactor chambers and a representative of the manufactured catheters. The cross-section of the chambers demonstrates the placement of the ventricular catheters in each type of chamber. The inlet and outlet of the chambers are also marked on the cross-sectional view

In total, four types of interchangeable bioreactor chambers (chambers) were designed and manufactured in this study. All chambers were modeled in Autodesk Inventor 2020 Pro (Autodesk. Inc, USA) based on prior work and with an objective to incorporate different generalized fluid flow directionality depending on catheter location within the ventricles. Chamber materials were investigated in a pilot study that led to the development of two types made of resin (single-inlet and a “dome” with multi-directional inlets), one of Polydimethyl Siloxane (PDMS, silicone), and one out of Polyethylene Terephthalate Glycol (PETG). These chambers were designed to incorporate protein, single cells, cell monolayers, multilayers, or co-cultures, and viable tissue structures. (Fig. 2).

Resin chambers were manufactured using an Anycubic Photon 4 K MSLA printer (Anycubic Technology CO., Limited, Hong Kong). The resin chambers (*n* = 45) were divided into two broad subcategories single inlet chambers for unidirectional flow (Resin) and multidirectional inlet chambers (Dome). Four iterations of the single inlet resin chambers with 0°, 45°,90°, and 135° inlet angles relative to the ventricular catheter were developed and printed to simulate CSF flow mixing specific to catheter positioning relevant to CSF bulk flow or pulsation production (*N* = 10/ per inlet angle) (S. Figure 2).

The high-throughput silicone chambers were modeled based on bioreactor chambers utilized in our previous in-vitro investigations where the incorporation of proteins and single cells in suspension or attached to the catheter surface was imperative [10, 11, 19]. The silicone chambers were assembled using luer-type barb connectors on the inlet and outlet ports, silicone tubing, and a glass outer shell. Forty chambers were manufactured in batches of 10. Each batch was contained in a custom holding tray (Fig. 2).

High-throughput Polyethylene Terephthalate Glycol (PETG) chambers were specifically designed for the incorporation of cell layers, tissue, or tissue-like structures for investigation of cell attachment and migration easily identifiable with real-time confocal and brightfield microscopy techniques without deconstructing the setup. These chambers were fabricated utilizing a vacuum forming machine (A3, Vacucu 3D, China) where a 3D printed resin mold was employed to transfer the chamber’s geometry onto transparent PETG sheets. The geometry of the resin mold was iteratively modified based on computational Fluid Dynamics (CFD) analysis to mimic CSF flow patterns through the catheter holes in enlarged, fixed ventricles. Overall, 50 PETG chambers were manufactured for this study. All the manufactured chambers were visually inspected post-processing for potential malformations and geometrical defects using a Trinocular stereo zoom microscope (SM-4TZ-144 A, AmScope, USA) with a 0.5X Barlow lens.

Flexible MRI-based models of patients’ ventricular systems were produced in three stages: filling, rotational molding, and demolding. Anycubic Kobra Max Fused Deposition Modeling printer (Anycubic Technology CO., Limited, Hong Kong) was utilized to produce a three-dimensional mold of the patient ventricular systems. The molds were designed such that the internal volume of the mold matched the volume of the patient ventricular system. In total 50 mL of uncured silicone was injected into the hollow molds through a 12-gauge stainless steel needle. The ventricles were exposed to biaxial rotation for three hours followed by an additional eighteen hours of stationary rest to complete the curing process. After the completion of the curing process, the molds were gently removed to expose the silicone ventricle model. AIMS was utilized to inject 165 mL, 26 mL and 8mL of degassed water into the ventricle model to simulate enlarged, moderately enlarged and slit-like ventricular systems respectively. The ventricles model was cut open with a scalpel and the material thickness was measured in ten spots using digital calipers (01407 A, Neiko, Taiwan).

### Computational fluid dynamics

**Fig. 3:**
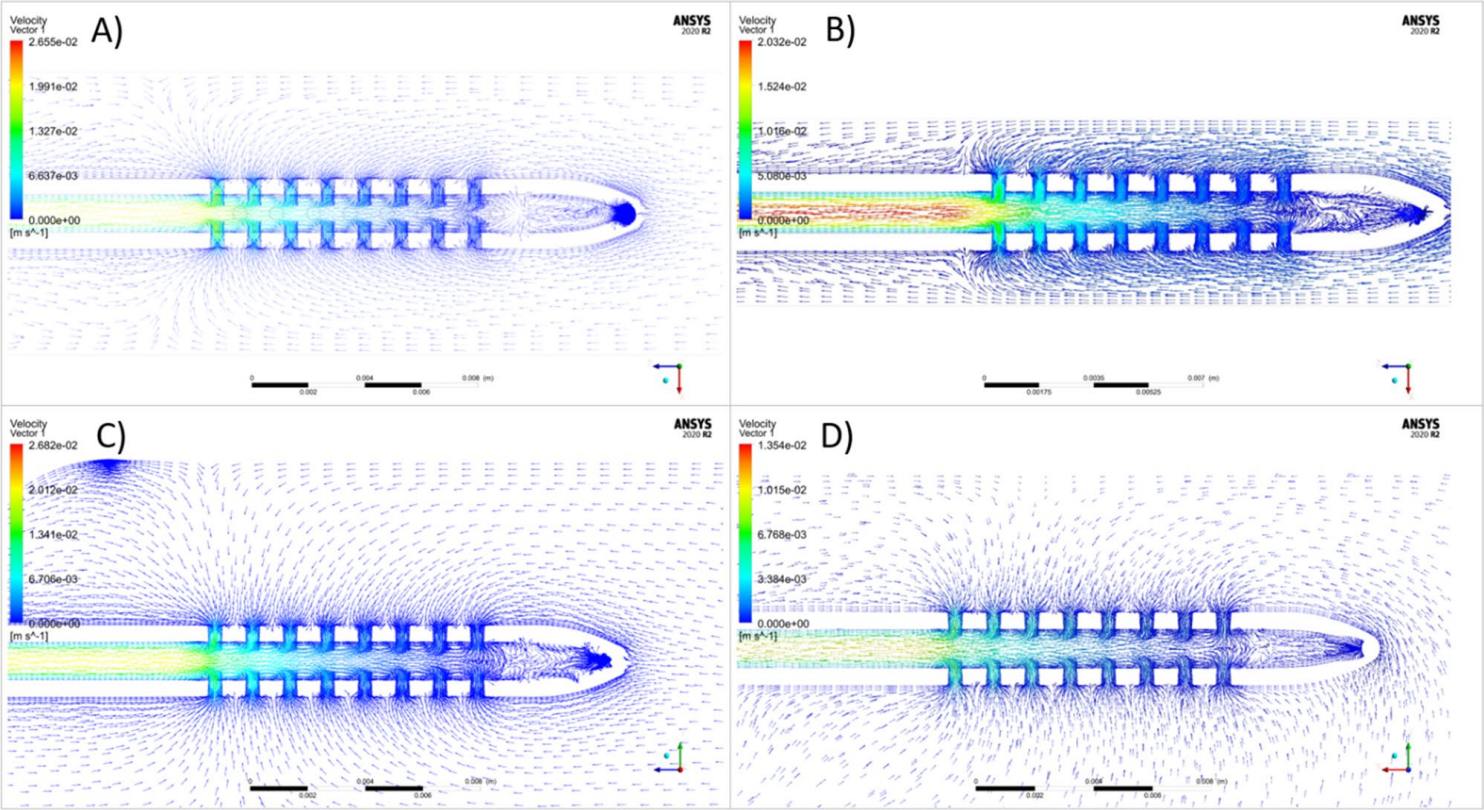
Visual representation of flow vector directionality in the vicinity of a commercial ventricular catheter with eight lateral holes per row (**A**) Single Inlet Resin Chambers-Inlet Angle 0 (**B**) High throughput silicone chambers (**C**) Dome Chambers (**D**) High throughput PETG chambers

The composite geometry in the Standard for the Exchange of Product Data (STEP) format was imported into ANSYS Fluent computational fluid dynamic software and was discretized into a nonuniform unstructured rigid grid. Finite element grid refinement was performed at the catheter drainage holes, lumen, and the inlet and outlet boundary face for each chamber variation. Adaptive uniform boundary layers were applied on all rigid faces of the computational model. Polyhedral mesh elements were used to capture curvature and proximity with the flow field regiment at the entrance of each drainage hole. Final mesh skewness ranged from 0.35 to 0.40 based on the bioreactor model simulated. In Fluent a laminar flow model was selected, and the kinematic viscosity of water was set equal to $\:0.75\times\:{10}^{-6}\:\frac{{m}^{2}}{s}$ [21]. A non-slip flow condition was applied at the walls of the computational domain. The inlet boundary conditions were set to match both the clinical measurement of pulsatile CSF outflow through External Ventricular Drain (EVD) in the pre-and post-operation state. A constant zero pressure was specific at the outlet. A transient solver using the Navier-Stokes equations was selected to simulate a full cardiac cycle of 1 s. Vector velocity profiles were captured in the chamber and catheter domains using Fluent Post Processing to observe different velocity flow fields with respect to the reactor model (Fig. 3, S. Figure 3).

### The control unit, reciprocating positive displacement pumps, and the fluidic components

The Control Unit consisted of an Arduino-based controller board and a custom-built, proprietary Python program with a custom user interface. The Control Unit interfaced with up to ten Reciprocating Positive Displacement Pumps (pumps) simultaneously, assembled according to our previously published study [17, 20]. Briefly, five 3-ml syringes in each pump were actuated forwards and backward by a drive unit to induce flow. The pumps acted as intermediaries between the fluidic components and the computer software. Thus, the pumps interfaced with a stopcock or a series of check valves (Duckbill Check Valve, Qosina, USA) through silicone tubing. The pumps, check valves, bioreactor chambers, and flow sensors (SLF3s-1300 F Series, Sensirion, Switzerland) were all placed in series in a circuit; flow instigated by the pumps traveled through the connected components and eventually drained into a separate reservoir (Fig. 1). AIMS was designed such that each one of the 50 pump channels was independent of the other bioreactor chambers. Specifically, there was no exchange of solution or flow interference between adjacent channels. The AIMS setup’s automated priming feature facilitated air bubble removal and constant fluid contact throughout the experiments. During the priming process, saline flowed through the chambers continuously for twenty minutes. The displaced air in the fluidic components was released in the reservoir and was forced out of the circuit through a filter with 0.22 $\:\mu\:m$ pore size.

AIMS was developed to recapitulate a wide range of physiologic and pathophysiologic CSF flow patterns with varying pulse amplitude (0.05-55 $\:\frac{mL}{min}$), pulsation rate (1-400 $\:\frac{Beats}{min}$), and bulk flow rate (0.01-5 $\:\frac{mL}{min}$) [17]. These variables were specified in the user interface to match clinical CSF flow measurements. The Python program transformed the user specifications into real-time commands that manipulated the pump’s output. Flow data were collected using the Sensirion USB Sensor Viewer program on a Hewlett-Packard desktop computer running Windows 10Pro operating system.

### Capacity and consistency analysis

In addition to flow simulation capabilities, AIMS was also utilized as a modular platform for chamber testing and quality control (S. Figure 1A-C). A pressure manometer (Extech HD700 Differential Pressure Manometer − 2PSI, Teledyne FLIR LLC, USA) was utilized to quantitatively analyze the capacity of the manufactured chambers under differential pressure. Each chamber underwent ten pressurization cycles, with the maximum gauge pressure reaching 140.7 $\:cm{H}_{2}O$. (13.8 kPa) (S. Figure 1A). The pressure was induced by the slow forward motion of the reciprocating pumps. The pressure curve was visualized and recorded via HD700 Data Acquisition software (Teledyne FLIR LLC, USA) during and after pressurization. The pressure curves were closely monitored in real-time and post hoc for signs of sudden depressurization or pressure loss due to a leak or chamber rupture. Overall, 1350 maximum pressure cycles were recorded (*N* = 10, 135 individual chambers).

In an alternative configuration of the setup, the absolute maximum amplitude induced by the AIMS at a 0.3 ml/min output volume rate was investigated (S. Figure 1B). A flow sensor was placed immediately after the stopcock. This experiment was also repeated with check valves to investigate the dampening effects of the valves. A similar setup was utilized to induce high amplitude (60 BPM, 0.34 ml/min bulk flow rate) pulsatile flow and to investigate the fluidic consistency of the pumps and the chambers. The pulsation rate (60 $\:\frac{Beats}{min}$) and bulk flow rate ($\:0.34\:\frac{mL}{min}$) were selected for their physiologic relevance to CSF production, while the peak amplitudes (50 $\:\:\frac{mL}{min}$) were chosen to maximize system volatility and to “stress test” the setup and the chambers [22]. A baseline of the pulsatile output was obtained by recording 10 separate 1-minute measurements of the pump output. The peak amplitude of ten consecutive peaks was analyzed per recording (*N* = 10, 10 measurements).

All high-throughput chambers, Single inlet Resin chambers (*N* = 40), silicone chambers (*N* = 40), and PETG chambers (*N* = 50) were manufactured in batches of 10 chambers and were analyzed with their respective batches as independent product lots. The peak amplitudes of batches were statistically compared across chamber types. The fluidic consistency of five individual Dome chambers was also investigated (*N* = 5). Overall, 1,450 peak amplitude data points were recorded with the flow sensors (S. Figure 1C). As previously reported, check-valves were essential to the automatic refilling functionality of AIMS. Therefore, check valves replaced the stopcocks for the remainder of the experiments.

### Long-term fluidic performance

The consistency of the AIMS setup’s volumetric output was evaluated from 0 to 30 days. The volumetric analysis of bulk output was performed by weighing the output at 0, 15, and 30-day intervals using analytic balance Mettler Toledo AT261 DeltaRange Analytical Balance (Mettler-Toledo, LLC, USA). AIMS was programmed to run a pulsatile 70 BPM, 0.3 ml/min profile uninterruptedly for the 30-day duration. A total of eight ten-minute weight measurements were collected across 15 pump channels per volume profile (*n* = 15) at each time point yielding 360 ten-minute measurements. The output of each channel was collected and weighed in individual beakers (S. Figure 1D). The pump output flow pattern was also recorded at each time point.

### Flow simulation and compliance

Compliance (C) was defined as the change in volume (V) relative to the change in pressure (P). As previously reported, the compliance of inline fluidic components such as the chambers ($\:{C}_{Bioreactor\:Chambers})$ had an impact on the amplitude and the waveform of the individual pulsations. A known volume of air was added to the pumps to augment the compliance ($\:{C}_{Augmented})$ in the circuits to better recapitulate the flow waveform of clinical measurements. This air volume was entrapped within the reciprocating portion of the pumps that were specifically accounted for in the program, thereby influencing the compliance of the system without interacting with the chambers throughout all experiments. The combined impact of augmented compliance and the inherent compliance of inline fluidic components ($\:{C}_{Total})$ on peak amplitude and pulsatile flow profile was investigated; to this end, 10 consecutive pulsations were recorded at varying amounts of augmented compliance (56.25 µL, 112.5 µL, and 225 µL air volume). Waveforms from chambers with varying $\:{C}_{Augmented}$ were aligned and transposed to demonstrate the impact of $\:{C}_{Augmented}$ on waveform profile in each chamber type. Additionally, the flow profile of the setups with augmented compliance was compared to circuits with no augmented compliance (0 µL).

$$\:{C}_{Total}\cong\:{C}_{Bioreactor\:Chambers}+{C}_{Augmented}$$

In a subset of samples, the CSF outflow from a pediatric patient’s EVD was referenced and then simulated via AIMS with all four chamber types independently [23]. An overlay of these data was used to compare the pulsations with total compliance adjusted. Pre-operation (pathophysiologic) and post-operation (physiologic) flow patterns were simulated with augmented compliance and without augmented compliance in Fig. 1A setup. The volume of air was iteratively adjusted for each chamber type to replicate the clinical flow pattern measurements as closely as possible. To match the temporal scale of the clinical measurement, a ten second segment of the pre-operation and post-operation flow waveform was represented in each chamber type with and without augment compliance.

In a subset of samples, an unused commercially available adjustable shunt valve was added to the setup to simulate the measurements of a noninvasive wireless monitoring device that measured CSF flow through shunt systems of high flow and low flow patients (S. Figure 1E) [24]. The shunt valve setting was manually adjusted as necessary prior to flow recapitulation. To match the temporal scale of the clinical measurement, a 150 s segment of waveform for high flow case and a 300 s segment of waveform for low flow case was visually represented. Additionally, a custom flow profile was produced to replicate the in-vivo measurements with the pumps directly. In this setup, the check valves were replaced with a stopcock, and the shunt valve was removed. The in-vivo measurements were replicated with the pumps directly using a custom profile; effectively replicating the combined impact of physiologic input and the mechanical action of shunt valves. This unique capability leveraged the setup’s programmability to produce complex, non-heterogenous flow patterns. This feature was also utilized to simulate clinical measurement of aqueduct flow patterns in a healthy volunteer and a hydrocephalic patient [25]. A 1600 millisecond segment of flow rate waveform was visualized to match the temporal scale of the clinical measurement.

Finally, a Watson Marlow 323 DU peristaltic pump (Wilmington, USA) equipped with a 314MC attachment and 0.38 mm bore/1.02 mm bore peristaltic tubing was utilized to investigate the compatibility of the bioreactor chambers with alternative sources of flow induction such as those from peristaltic pumps (S. Figure 1F). This setup was used to simulate the EVD pre-recovery and post-recovery flow patterns simulations with all types of bioreactor chambers and was compared to the same output from AIMS.

### Biocompatibility

The biocompatibility of chamber materials was considered by quantifying the growth of human astrocytes on each material type over five days. To assess the biocompatibility of resin, silicone, and PETG, a puck with a surface area equivalent to 50% of a 24-well plate bottom was secured to the bottom of the respective wells using a silicone-based adhesive. In total, eight wells within a 96-well microplate were prepared for each experimental condition: (1) negative control (formaldehyde), (2) neutral (uncoated well-plate), (3) Resin, (4) silicone, (5) PETG, (6) Silicone-based glue.

Initial cell count was made using a hemocytometer and a trypan blue exclusion assay. A media stock solution was prepared by combining astrocyte media (ScienCell, USA), supplement kit, 10 mL fetal bovine serum (FBS), 5 mL penicillin/streptomycin, and 5 mL astrocyte growth serum, (ScienCell, USA), and an additional 10 mL FBS (ScienCell, USA). After the 5 days, a 2.5 mg/mL aliquot of DiD was diluted to a 20 µg/mL solution using astrocyte media, and a 5mM stock solution of pluronic F127 was diluted to a 100 µM solution using stock media. After removing existing media from wells, the wells were rinsed with 50uL of Hank’s Balanced Salt Solution (HBSS). Then, a 20uL of DiD and 20uL pluronic F127 solution were added to each well. The samples were incubated for 2 h. This process was repeated three times. The wellplates were imaged on a Leica TCS SP8 confocal laser scan microscope using a Leica 10× magnification dry lens.

### Imaging and optical output

Fluorescent particles, specifically Green Fluorescent particles (G0500, Thermo Fisher Scientific, USA), characterized by a diameter of 5 μm and a density of 1.06 $\:\frac{g}{mL}$, were introduced into a 3 mL saline solution. The flow rate was set to post-operation clinical measurement of CSF flow in a pediatric patient [23]. These particles were subsequently directed into a chamber for observation using Resonance scanning confocal microscopy (RS-G4), installed on an upright microscope platform (Caliber ID, Andover, MA, USA). The imaging protocol utilized a wavelength of 488 nm to ensure stable signal acquisition from the fluorescent microspheres as they traversed the flow field proximal to the drainage holes. Single z-stack frames from a 5-second recording of microsphere movement near each lateral hole were exported. The pictures were sequenced and overlapped to produce long exposure views of microsphere motion near the lateral holes of unused and patient-explanted catheters from our biorepository.

Six digital microscopes (Plugable Technologies, USA) were utilized to construct an imaging setup to produce brightfield microscopic and macroscopic time-lapses of the PETG chambers in a high throughput manner. The setup also featured a custom-built chassis that held up to 24 chambers in total, and a linear actuation mechanism that interfaced with the chassis and propelled the chambers forward such that the next row of catheter holes could be imaged. This process continued until all 24 chambers were imaged. An ultraviolet (UV) light source and fluorescent dye were utilized to record a macroscopic view of the fluorescent dye entering through the inlet of the PETG chamber and leaving through the ventricular catheter.

### Statistical analysis

Statistical analyses were performed using SPSS Statistics Suite (IBM Corporation, USA). The Levene Test of Homogeneity of Variances was performed to evaluate the homoscedasticity of the datasets. Parametric data were analyzed using One-way analysis of variance (ANOVA) or repeated measures ANOVA tests. Non-parametric data were analyzed using Kruskal-Wallis test. A linear regression analysis was performed as a simple predictive analysis to investigate the correlation between variables and the coefficient of determination ($\:{R}^{2}$) was calculated. For all tests, a confidence interval was set at 0.95 (α = 0.05) A *post hoc* Tukey test was performed when the null hypothesis (no difference in the group means) was rejected.

## Results

**Fig. 4:**
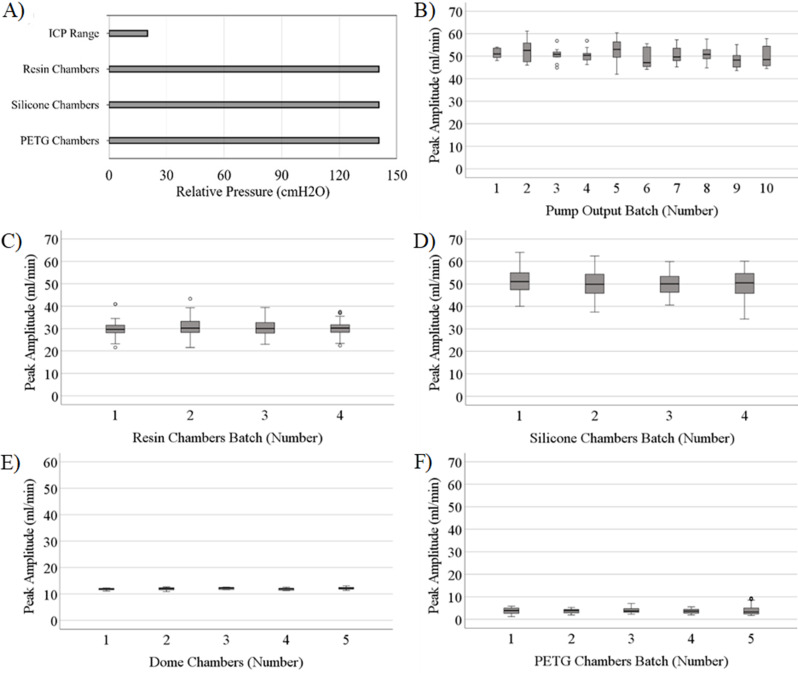
Capacity and consistency across batches of bioreactor chambers (**A**) The pressure domain of bioreactor types relative to intracranial pressure (ICP) physiologic range; All chamber types withstood repeated pressurization cycles to 140.7 $\:cm{H}_{2}O$ gauge pressure: significantly beyond the reported ICP domain in most hydrocephalus patients. (**B**) The peak amplitude of ten consecutive peaks induced by the pumps at 60 BPM pulsation rate, and 0.34 ml/min bulk flow rate. 60BPM and 0.34 ml/min were selected for their physiologic relevance to CSF production, while 50 ml/min peak amplitude waves were chosen to maximize system volatility and to “stress test” the setup and the chambers [22]. (**C**) The peak amplitude of 40 single inlet resin chambers manufactured in four batches (*N* = 10 per manufactured batch); (**D**) the Peak amplitude of 40 silicone chambers manufactured in 4 batches (**E**) The peak amplitude of five dome chambers manufactured in a single batch (**F**) The peak amplitude in 50 PETG chambers, manufactured in 5 batches of *N* = 10 per manufactured batch. Open circles indicate outliers in peak amplitude measurements

Overall, 135 chambers were manufactured and tested in this study (Fig. 2). No significant manufacturing deformities were detected. All chambers withstood 10 pressurization cycles to 140.7 $\:cm{H}_{2}O$ gauge pressure: significantly beyond the reported ICP domain (0–30 $\:cm{H}_{2}O$) in most pediatric and adult hydrocephalus patients (Fig. 4A) [26]. No leaks or sudden ruptures were observed during the pressurization cycles. These results indicated that all the manufactured chambers were suited for further investigations of peak amplitude consistency and flow simulations.

Computational fluid dynamics revealed flow directionality given by vector flow patterns in the four-chamber types (Fig. 3, S. Figure 3). While the velocity magnitude slightly varied between different types of chambers, a general gradient in flow velocities was observed such that the lowest flow velocity was observed near lateral holes closest to the catheter tip, and the highest flow velocity was observed near lateral holes furthest from the catheter tip. Flow directionality at the catheter-CSF interface aligned with clinical conditions observed in computational fluid dynamics models within patient ventricles modeled from patient imaging (preliminary data not reported).

### Peak amplitude consistency analysis

The AIMS’s output peak amplitude in the experimental setup with stopcocks was 65 ml/min while the setup incorporating check valves reached a maximum peak amplitude of 27.26 ml/min (S. Figure 4). No statistically significant difference was observed between the peaks produced by AIMS over time and overall batches (*P* = 0.36) (Fig. 4B). Similarly, peak amplitudes through four batches of resin chambers were analyzed (Fig. 4C) with no significant difference observed between the four batches of single inlet resin chambers (*P* = 0.09). The peak amplitude observed in the four batches of silicone chambers demonstrated a similar trend such that no statistically significant difference was observed in the peak amplitudes recorded across the four batches of silicone chambers (*P* = 0.51) (Fig. 4D). Similarly, no statistically significant differences were observed in the peak amplitudes conducted through five dome chambers (*P* = 0.23) (Fig. 4E). Finally, no statistically significant difference was observed between the peak amplitudes conducted through 5 batches of PETG chambers (*P* = 0.48) (Fig. 4F).

While the pump input into all chambers remained constant, the differences in the inherent compliance of the chambers had an impact on the recorded output peak amplitude (Fig. 4). The overall mean peak amplitude for the pumps was 50.58 $\:\frac{mL}{min}$. The mean peak amplitude for resin and silicone chambers was 30.26 $\:\frac{mL}{min}$ and 50.29 $\:\frac{mL}{min}$ respectively. The mean of peak amplitudes across dome chambers was 12 $\:\frac{mL}{min}$. Interestingly, 3.77 $\:\frac{mL}{min}$, the lowest mean of peak amplitudes was observed in PETG chambers while the smallest standard deviation was recorded in Dome chambers (S. Table 1).

### Compliance

**Fig. 5:**
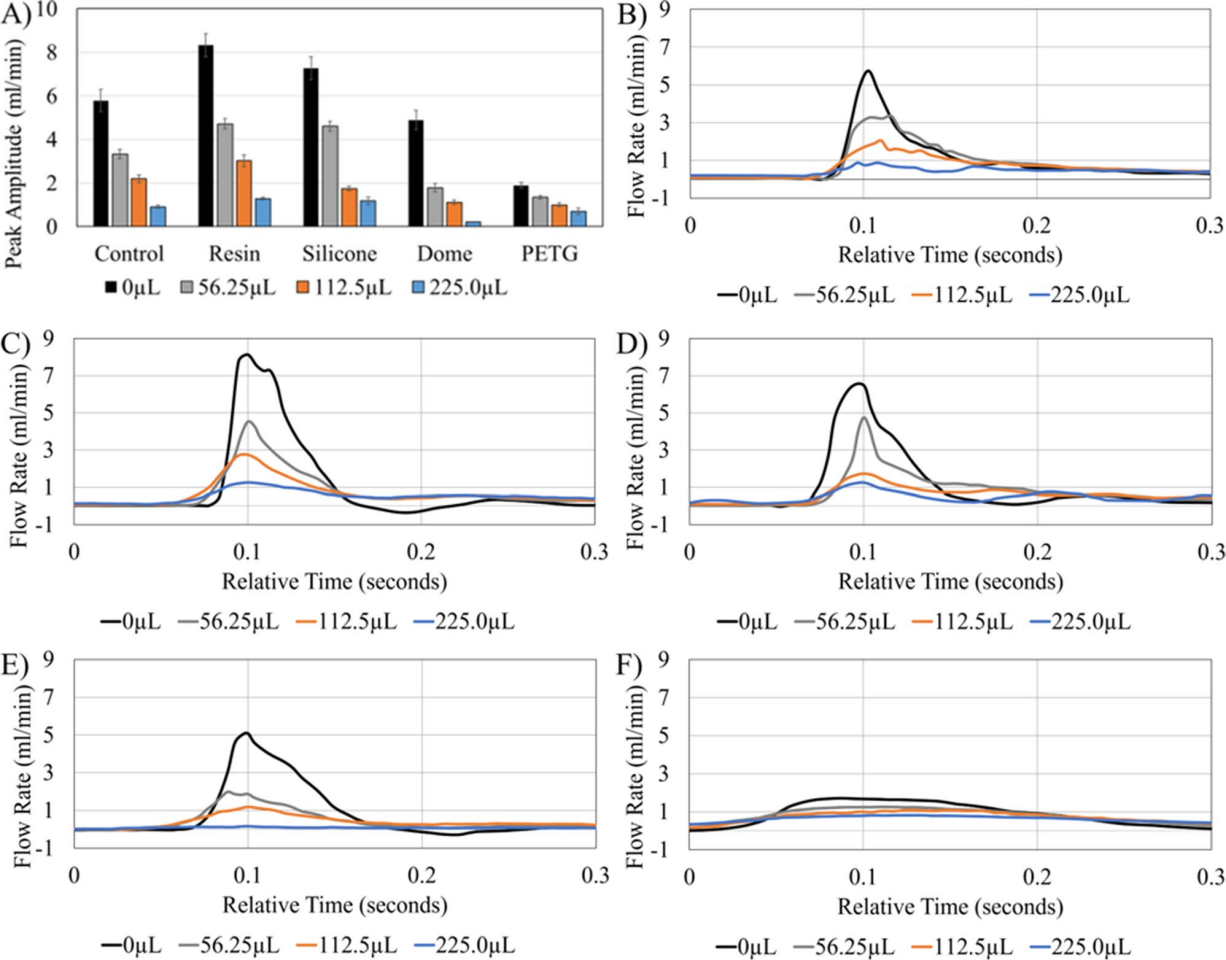
The impact of augmented compliance on peak amplitude and flow waveform. (**A**) Average Peak amplitude relative to chamber type and volume of augmented compliance, (**B**) Representation of pump output (control) waveform with augmented compliance flow pattern overlay, (**C**) Representation of waveform in single inlet resin chambers with augmented compliance flow pattern overlay (**D**) Representation of waveform in silicone chambers with augmented compliance flow pattern overlay (**E**) Representation of waveform in dome chambers with augmented compliance flow pattern overlay (**F**) Representation of waveform in PETG chambers with augmented compliance flow pattern overlay. The term “relative time” on the x-axis Fig. 5B-F indicates that the peaks of the waveforms from multiple measurements were aligned and transposed to demonstrate the impact of augmented compliance on the waveform

The investigation AIMS output peak amplitude at 0 µL (no augmented compliance), 56.25 µL, 112.5 µL, and 225 µL air volume demonstrated a reverse correlation between the air volume and pulse amplitude (Fig. 5). A similar trend was also observed in circuits with chambers between the peak amplitude and the air volume across all measurements. A higher amplitude was measured in the outlet of Resin chambers and silicone chambers compared to the pump output. In contrast, a lower amplitude was observed in Dome and PETG chambers (S. Table 2). A smoother, more gradual waveform was observed as a result of increased total compliance (Fig. 5B-F).

**Fig. 6:**
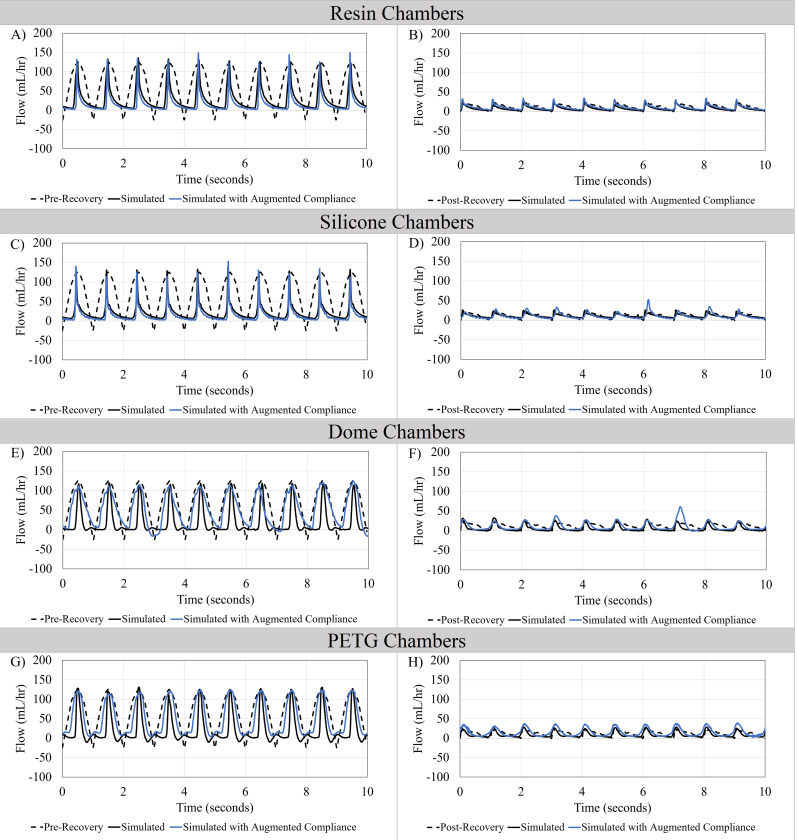
Recapitulation of clinical measurements of CSF Flow through an External Ventricular Drain (EVD) in a pediatric patient pre-recovery and post-recovery. Clinical measurement of one repeated CSF flow waveform through an EVD of a pediatric patient was simulated with and without augmented compliance in all four chamber types. (**A-B**) Resin Chambers, (**C-D**) Silicone Chambers, (**E-F**) Dome Chambers. (**Fig. 6A, C, E, G**) demonstrate the Pre-recovery (pathophysiologic) and (**Fig.** **6B, D, F, H**) demonstrate post-recovery (physiologic) flow patterns

An overlay of outflow waveforms from an EVD in a pediatric Chiari patient and from our pump is shown in Fig. 6. The pathophysiologic measurements (pre-recovery) and physiologic measurements (post-recovery) were simulated in four-chamber types. The peak amplitude of the pulsatile flow conducted in all four types of chambers matched the amplitude of pre-recovery measurements. However, the flow patterns observed in PETG and Dome chambers with augmented compliance best fit the pre-recovery EVD measurements (Fig. 6A, C, E, G). In contrast, flow curves and amplitudes presented in post-recovery simulations closely matched a clinical repeated waveform in all chamber types (Fig. 6B, D, F, H).

**Fig. 7:**
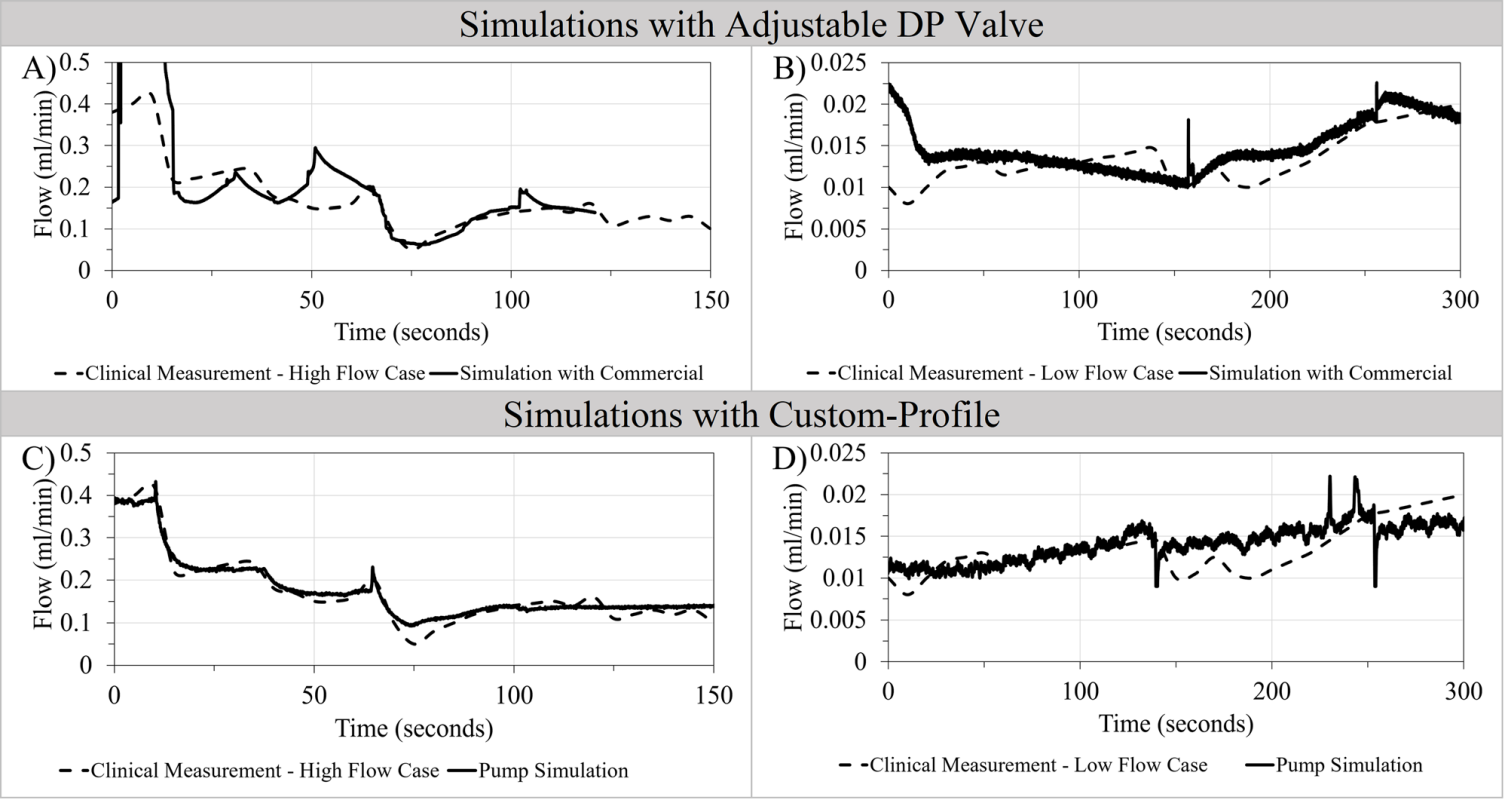
Recapitulation of CSF flow measurements through a shunt system in hydrocephalus patients with high flow rates and low flow rates using adjustable differential pressure (DP) valve and custom-profile feature of AIMS setup. Note that the presence of shunt valves in these clinical measurements had a significant impact on the CSF flow patterns (**A**) Recapitulation of CSF flow pattern observed in a hydrocephalus patient with high flow rate using the AIMS setup and a commercial differential pressure valve. Note that flow measurement was terminated around 120 s (**B**) Recapitulation of CSF flow pattern observed in a hydrocephalus patient with low CSF flow rate using AIMS setup and a commercial differential valve (**C**) CSF flow pattern in a hydrocephalus patient with high CSF flow rate using the custom-profile feature of the AIMS setup directly without a DP valve. (**D**) Recapitulation of clinical measurement of CSF flow pattern in a hydrocephalus patient with low CSF flow rate using the custom-profile feature of the AIMS setup directly without a DP valve

The flow waveforms in Fig. 7 demonstrate the measurement of CSF flow patterns in a hydrocephalus patient with high CSF flow rate (Fig. 7A, C) and a hydrocephalus patient with low CSF flow rate (Fig. 7B, D). The simulations using the custom-profile feature of the AIMS setup matched the amplitude and the waveform of clinical measurements of high flow case and low flow case compared to simulations with an adjustable differential pressure (DP) valve. The custom-profile feature of the setup was also utilized to recapitulate CSF flow in the aqueducts of a healthy volunteer and a hydrocephalic patient (S. Figure 5).

**Fig. 8:**
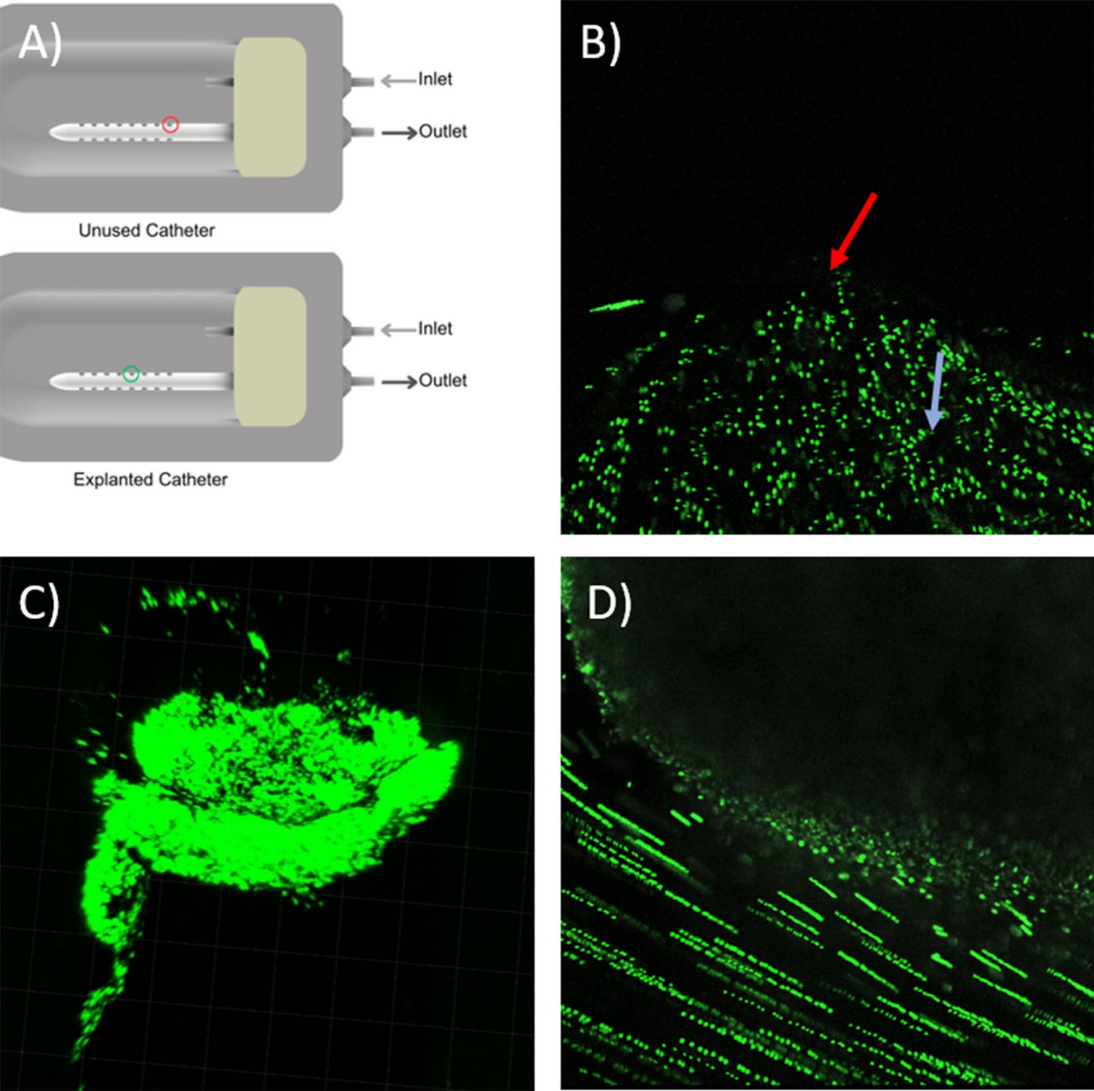
Representative visualization of flow through the lateral holes of unused and explanted ventricular catheters in a PETG chamber. (**A**) Schematic of an unused commercial catheter and an explanted catheter in PETG chambers. The lateral hole furthest from the tip is indicated by the red circle. The green circle identifies the location of tissue aggregates on the explanted catheter (**B**) Five-second long exposure view of microspheres movement into the lateral hole furthest from the catheter tip in a single focus layer. The red arrow indicates the location of the lateral hole. An example of a florescent microsphere is indicated with the blue arrow (**C**) Three dimensional view of tissue aggregates on an explanted ventricular catheter from Harris Lab shunt bank in a PETG chamber (**D**) Five-second long exposure view of florescent microsphere movement around tissue aggregates on the lateral hole of an explanted catheter in a single focus layer

The fluorescent microspheres visualized the flow fields proximal to the lateral holes of unused and explanted catheters as they traversed through PETG chambers (Fig. 8). The long-exposure view of the particle movements into the lateral holes of the unused ventricular catheter revealed enhanced microsphere flow through lateral holes furthest from the catheter tip compared to lateral holes closest to the catheter tip (Fig. 8B). These results were consistent with CFD simulations in Fig. 3D and previous studies. In a separate PETG chamber, the tissue aggregates on an explanted ventricular catheter were successfully imaged (Fig. 8C). The z-stack analysis of the tissue aggregate revealed the three-dimensional geometry of tissue aggregates on a lateral hole of an explanted catheter. While the flow of microspheres through the lateral holes of unused catheters was evident in the long exposure images, no flow was observed through a lateral hole on an explanted catheter with large tissue aggregates in this single focus layer. (Fig. 8D).

### Long-term performance and biocompatibility

**Fig. 9:**
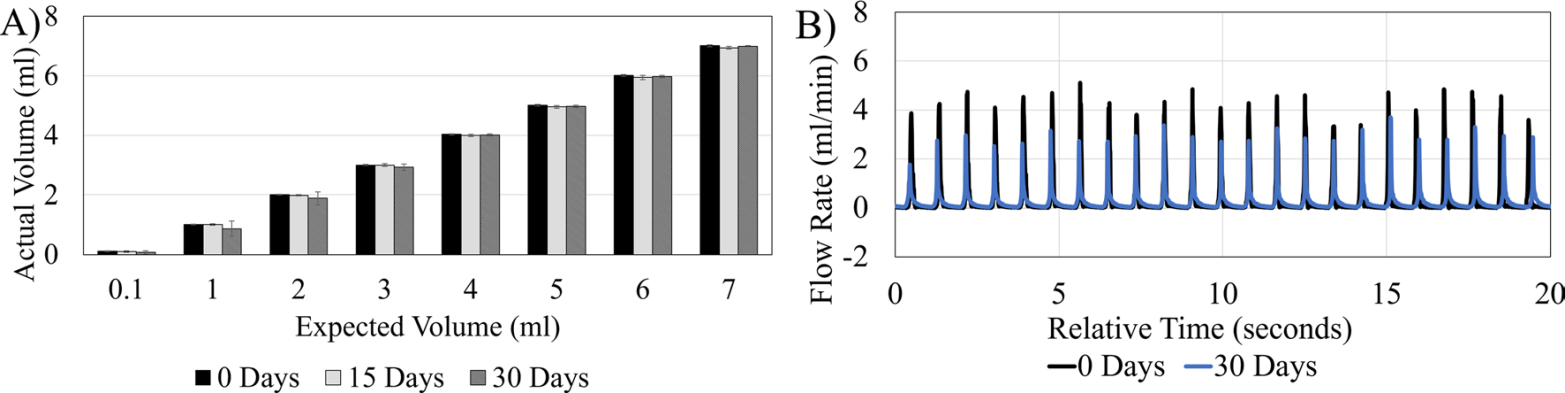
AIMS setup performance during 30 days of pulsatile flow (**A**) The volumetric output of the pump over 30 days +/- standard deviation. The pump bulk output remained consistent through the 30-day experiment (**B**) 20-second overlay of original and 30-day flow waveform output (70BPM, 0.3 ml/min bulk flow rate) representing the pulsatile flow pattern produced by AIMS setup before, and after 30 days. The pump output and pulsation rate remained consistent throughout the 30 days

The volumetric analysis of the AIMS setup’s consistency for long-term (up to 30 days) in-vitro studies indicated that the coefficient of determination ($\:{R}^{2}$) between the expected output volume and the measured volume was 0.9998, 0.9999, and 0.9992 for 0 days, 15 days, and 30 days measurements respectively. These results are comparable to the $\:{R}^{2}$ reported in our previous study (Fig. 9A). There were no statistically significant differences between the bulk flow output measurements at each timepoint (*P* = 0.999). The output flow pattern also demonstrated consistent beat rate before and after the 30-day run (Fig. 9B). However, the 30-day waveform amplitude was on average lower than the initial waveform.

**Fig. 10:**
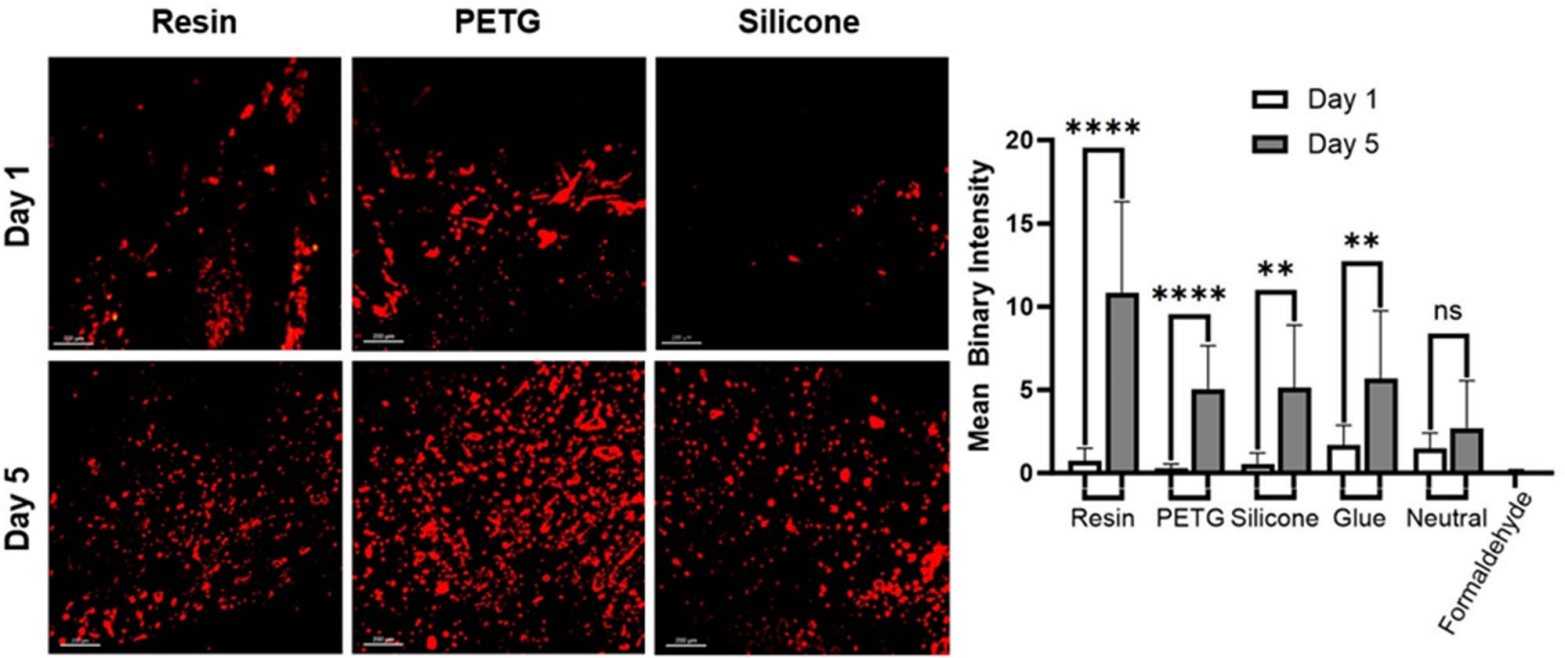
Investigation of chamber material biocompatibility with human astrocytes. The biocompatibility of chamber material type was investigated by quantifying the growth of human astrocytes over five days on each material type

In the investigation of chamber material biocompatibility with human astrocytes, a statistically significant increase was observed in the mean binary intensity of resin (*P* < 0.0001), PETG (*P* < 0.0001), Silicone (*P* = 0.0014), and silicone glue (*P* = 0.0016) over the 5-day incubation period as illustrated in Fig. 10. In contrast, no statistically significant increase was observed in the neutral control group (*P* = 0.1232). These compelling results underscored that the future investigation of foreign-body response in hydrocephalus patients with resin, PETG, and silicone chambers is warranted.

**Fig. 11:**
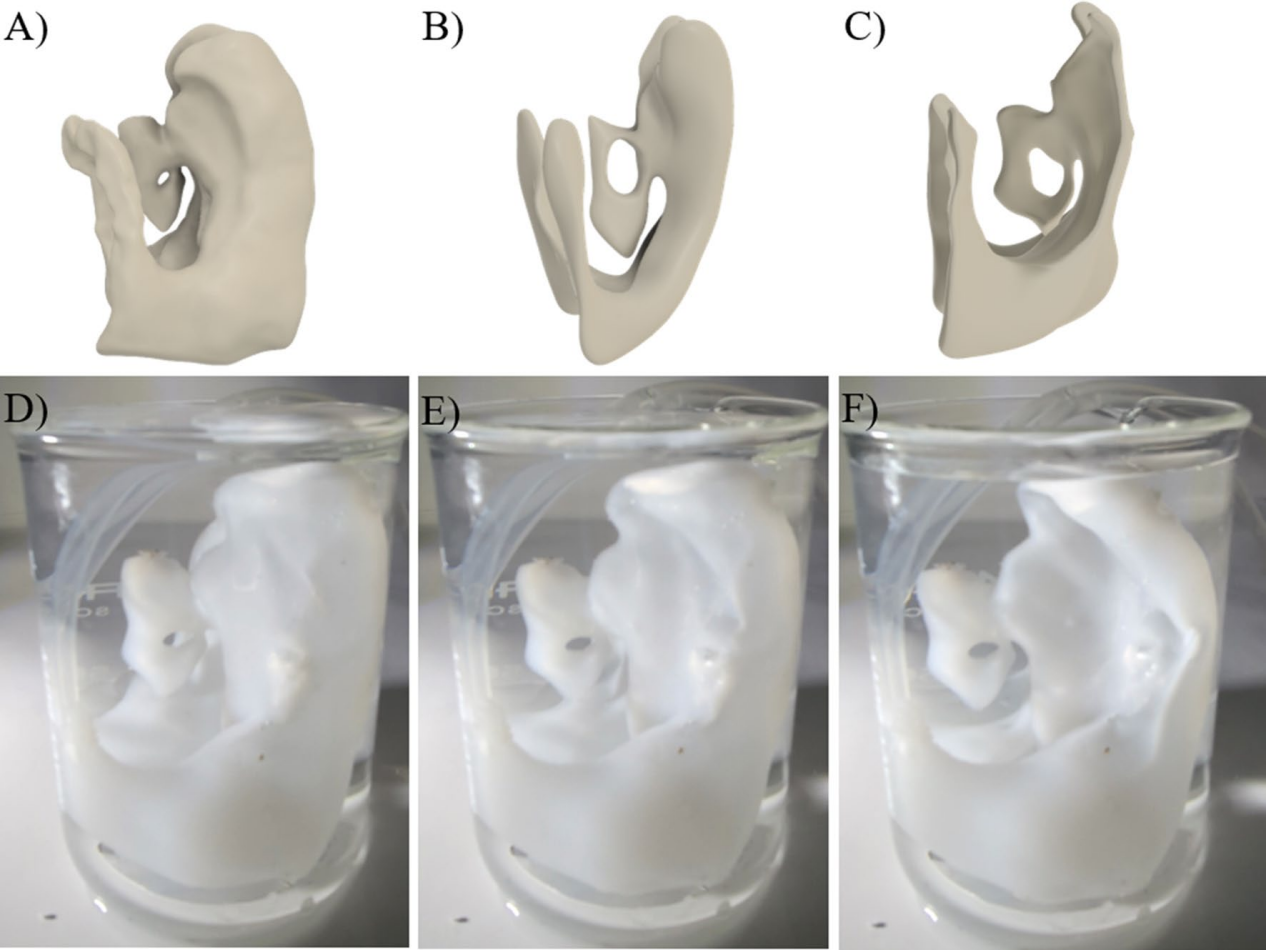
Flexible MRI-based ventricular system model. (**A**) Three-dimensional representation of enlarged ventricular system of a hydrocephalic patient. The internal volume of the ventricular system was 165 mL (**B**) Three-dimensional representation of moderately enlarged ventricular system of a hydrocephalic patient. The internal volume was 26 mL. (**C**) Three-dimensional representation of slit-like ventricular system of a hydrocephalic patient. The volume of was 8 mL **D** Flexible ventricular system model filled with 165 mL of saline to simulate the enlarged condition. (**E**) Flexible ventricular system model filled with 26 mL of saline to simulate the moderately enlarged condition. (**F**) Flexible ventricular system model filled with 8 mL of saline to simulate the slit-like condition

The dynamic change in the geometry of the ventricular system was visualized via the flexible MRI-based ventricular system model (Fig. 11). The ventricle model was successfully produced and was extracted from the mold. No leaks or ruptures were observed during the experiment. The thickness of the ventricle model measured 2.17 mm ± 0.67 mm. The flexible lateral ventricles of the model expanded similar to the enlarged ventricles of a hydrocephalus patient when injected with 165 mL of saline (Fig. 11A, D). The partial deflation of the ventricles was observed at 26 mL of saline, resembling the moderately enlarged ventricles of hydrocephalic patients (Fig. B, E). Finally, a complete collapse of the lateral ventricles was observed after an 8 mL saline injection. This observation was consistent with the MRI of a hydrocephalus patient with slit-like lateral ventricles (Fig. C, F). A gross visual comparison between the flexible MRI-based model and the MRIs of a hydrocephalus patient showed that the model generally replicated the mechanics of the lateral ventricles in a hydrocephalus patient. While these preliminary results demonstrated a proof-of-concept for producing anatomically accurate model of ventricles they also emphasized the need for a future study to further test and validate the models.

## Discussion

This study demonstrated the capabilities of AIMS as a modular, high-throughput testing platform for rigorous real-time data collection across as many as 50 concurrent channels for 50 simultaneous samples. All chamber types exhibited robust performance by withstanding pressure conditions beyond typical intracranial pressure levels without breaking down and/or leaking over time. Our analyses revealed no significant differences in peak amplitude among any batches of chambers, highlighting the consistency and reliability of the chamber fabrication and the AIMS setup.

The investigation of flow patterns through the chambers coupled with CFD analysis provided valuable insights into the impact of chamber design and material on pulsatile flow vectors for use in future hypothesis-driven investigations of environmental impact on ventricular catheter obstruction. Specifically, our analysis with CFD indicated that the single-inlet and muti-directional inlet resin chambers were best suited for producing chambers featuring complex shifts in CSF flow direction with similar results to catheters in hydrocephalic ventricles. This generalized approach would enable us to investigate how the catheter might perform in different clinical scenarios where the flow directionality could vary depending on the catheter’s position within the ventricles in future studies. Future work on these chambers will use these data and others to optimize for CSF patterns, flow velocities, and shear, and will address the source of pulsation to more directly mimic ventricles of varying degrees of ventriculomegaly.

The biocompatibility data point to all chambers being excellent modifiable and modular surfaces that can support the incorporation of cells and tissue. In addition, data indicate that silicone chambers provide a robust and more efficient platform for investigating interactions between CSF, individual cells, and shunt hardware in future studies. When and if future modifications are considered, the contact area between biological specimens and chamber materials should continue to be considered. The PETG bioreactor chambers, while not as fluidically complex as resin chambers or as user-friendly as silicone chambers, possessed the highest optical clarity compared to the other chamber types for repetitive analysis over time without opening the system and making it susceptible to bacterial contamination (Table 1).

While no statistically significant differences were observed within the batches of chambers in the peak amplitude analysis (Fig. 4), a larger variance in peak amplitude was observed in silicone and resin chambers. We hypothesize that the chambers with lower inherent compliance are more susceptible to peak amplitude variations such that slight variations in the chamber construction result in measurable changes in overall compliance and peak amplitude. Moreover, the investigation of compliance (Fig. 5) demonstrated compatibility of resin and silicone chambers with high amplitude pulsatile flow while PETG chambers’ relatively higher inherent compliance significantly reduced the peak amplitude of pulsations. These results may suggest that PETG chambers might be more suitable for high throughput investigations that require consistent peak amplitudes over a relatively narrow range, while silicone and resin chambers could be utilized to investigate the interaction between shunt devices, immune cells, and high amplitude pulsatile flow.

**Table 1:** **Comparison between the features of resin**,** silicone**,** and PETG chambers.** The resin chambers demonstrated the highest degree of flexibility in producing complex fluidic designs. Silicone chambers were the easiest chamber type to manufacture, sterilize, and use. PETG chambers had the highest inherent compliance and optical clarity compared to resin and silicone chambers

Features	Most Suitable Chamber Type
Fluidic Complexity and Design Flexibility	Resin Chambers **>** PETG Chambers **>** Silicone Chambers
Ease of Use	Silicone Chambers **>** Resin Chambers **>** PETG Chambers
Inherent Compliance	PETG Chambers **>** Resin Chambers **>** Silicone Chambers
Ease of Sterilization	Silicone Chambers **>** PETG chambers **>** Resin Chambers
Optical Clarity	PETG Chambers **>** Silicone Chambers **>** Resin Chambers

As presented in Figs. 3 and 5 chamber design and material had a significant impact on the measured amplitude and flow waveform. Nevertheless, AIMS replicated clinical measurements of physiologic and pathophysiologic CSF bulk flow, flow pulsation amplitude, and pulsation frequency in four distinct bioreactor chamber types utilizing code features and augmented compliance (Fig. 6). Previous studies primarily utilized peristaltic pumps to recapitulate pulsatile CSF flow [10, 11, 18]. The presented chambers were also compatible with a peristaltic pump. However, the output waveforms from the chambers with a peristaltic pump had limited relevance to the clinical measurements of CSF flow (S. Figure 6). Microcontroller based add-on systems to peristaltic pumps previously demonstrated the capability to regulate flow rate, pressure and amplitude by controlling the motor rotation rate and utilizing a series of pressure valves and sensors. However, AIMS capability to independently regulate bulk flow rate, amplitude and pulsation rate was required to recapitulate a clinical waveform for physiologic and pathophysiologic waveforms presented in this study [17, 20]. Future work will continue to augment AIMS such that individual waveforms from clinical cases can validate the flow velocity and shear through the ventricular catheter.

A limitation of EVD flow measurements is that the flow patterns do not reflect the impact of differential pressure valves on CSF dynamics in shunted hydrocephalus patient [23]. Figure 7 reiterated the important influence of DP valves on CSF dynamics and demonstrated the compatibility of AIMS with commercial shunt valves. Compared to EVD simulations (Fig. 6), flow patterns through commercial valves were significantly more heterogeneous, rapid, and erratic, especially in a referenced high-flow patient (Fig. 7) [24]. Variables such as inherent mechanical variations among shunt valves, limited adjustability, and unpredictable response to pulsatile flow significantly restricted the ability to simulate the in-vivo flow measurements using commercially- available shunt valves. The recapitulation of the in-vivo measurements of High-Flow and Low-Flow patients directly using the custom-profile feature of the AIMS setup addressed these limitations.

Another advantage of the custom-profile feature was the ability to conduct flow in the forward and backward directions; a hallmark of CSF flow in the aqueducts (S. Figure 5) [25]. Note that a higher amplitude was observed in the pathological condition, consistent with Fig. 6 flow patterns. The presence of retrograde flow in the ventricles and along the spinal cord has been previously reported [16, 25, 27, 28]. A secondary objective of S. Figure 5 was to also demonstrate the capabilities of AIMS to conduct retrograde flow. Future studies will investigate the impact of CSF flow directionality on astrocytes and macrophage proliferation and activation [29].

While statistically significant cell growth was observed in astrocytes exposed to the high throughput chamber materials (Fig. 10), the geometry and the volume of the chambers were significantly different from the dynamic geometry of the brain ventricular system. This measure was taken to achieve a high throughput testing platform based on the limitations of cell culture and benchtop experiments. However, previous studies have demonstrated the importance of anatomically realistic in-vitro models in the investigation of CSF dynamics [15, 16, 30–32]. To account for this potential oversight, we have fabricated flexible silicone reproduction of a pediatric hydrocephalus patient’s MRI (Fig. 11). In combination with AIMS setup, our flexible MRI-based models aim to both replicate the anatomical features of hydrocephalus patients ventricles, and to accurately reflect the complex mechanics of the lateral ventricles associated with the pulsatile flow of CSF through shunt systems. Although these phantoms might not be suitable for high throughput applications they might provide insights into overdrainage, underdrainage and shunt obstruction [33]. The model presented here is a proof-of-concept for such a dynamic phantom. These results warrant a future investigation to test and validate the model based on quantitative comparisons to MRI data of hydrocephalus patients, such as volumetric changes, nominal-actual comparison, FOHR and Evan’s index comparison and other methods.

**Fig. 12:**
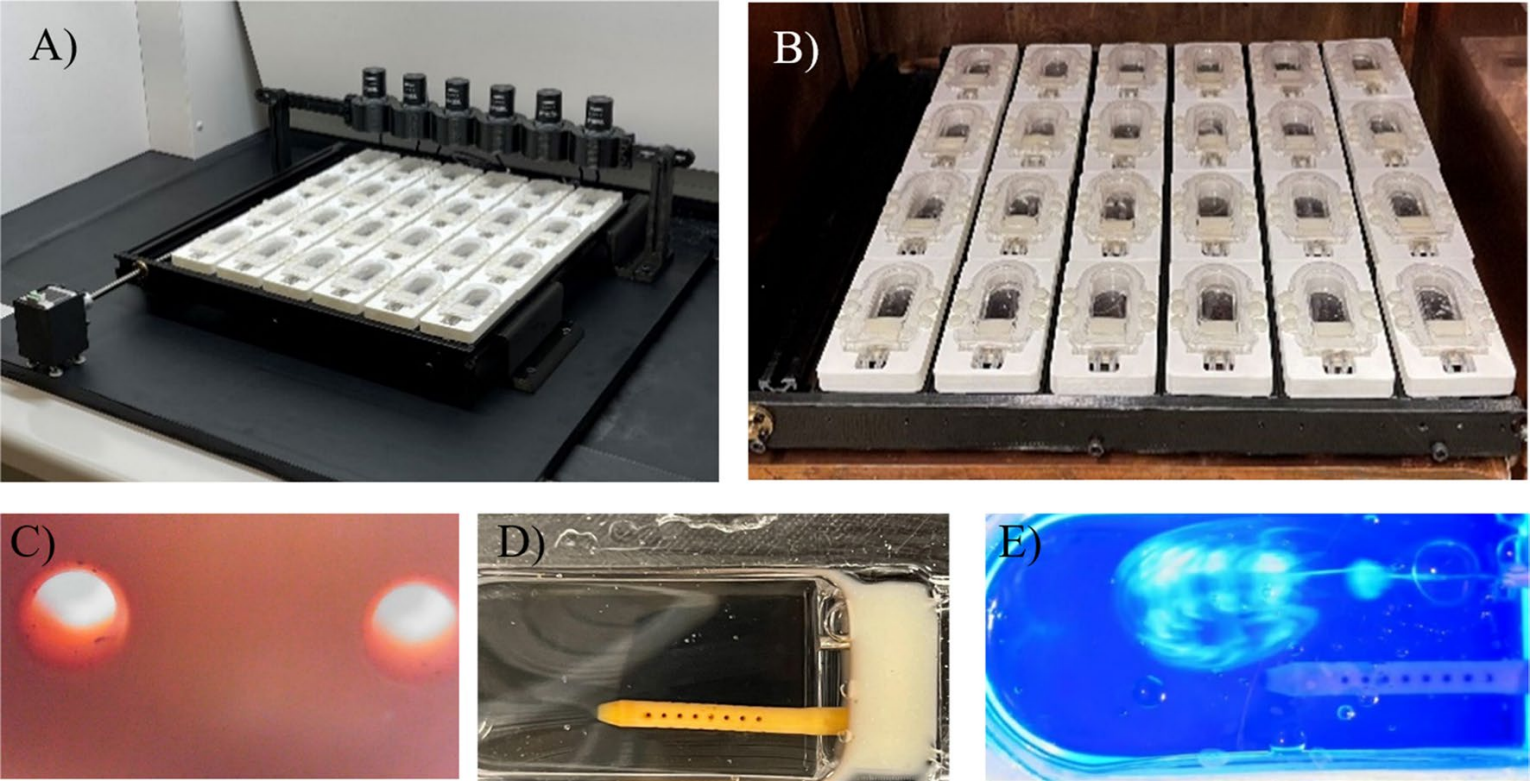
Custom-built High-throughput imaging setup for PETG chambers. (**A**) The setup was utilized to automatically produce a time-lapse of fluid flow through PETG chambers using brightfield digital microscopes. (**B**) The detachable chassis was specifically designed to fit on a standard incubator tray; suitable for experiments that require long-term incubation. (**C**) Brightfield view of individual lateral holes of a commercially available ventricular catheter in a PETG chamber (**D**) Brightfield view of the proximal end of a commercially available ventricular catheter in a PETG chamber. (**E**) The setup was utilized to produce detailed timelapse videos of fluid flow through ventricular catheters using UV light and florescent dye

Invaluable insights into the mechanisms of shunt obstruction were previously obtained using endpoint analysis techniques, highly detailed confocal images, and real-time visualization of flow vectors through unused and explanted catheters under physiologic and pathophysiologic flow conditions providing a paradigm shift in understanding the pathogenesis of shunt failure and improving treatment (Fig. 8) [34]. However, confocal imaging of more than one sample in real-time might be cumbersome in high-throughput studies. This limitation was addressed with the high-throughput imaging capabilities of the AIMS setup (Fig. 12) (S. Vid 1). Timelapses and real-time videos have been instrumental in identifying the pathogenesis of other neurological conditions. Future studies will utilize the high throughput imaging capabilities of the AIMS setup to investigate mechanisms of shunt obstruction. For instance, the high throughput capabilities of AIMS setup will be utilized to investigate the impact of CSF flow dynamics on protein dynamics. These capabilities may also be utilized in the investigation of proximal catheter obstruction during hematoma. Finally, the high-throughput chambers will be utilized in combination with previously reported three-dimensional hydrogel scaffold to investigate shunt obstruction precipitated by foreign body response and cell attachment [35].

## Conclusion

In this investigation AIMS improved upon our previous long-term in vitro systems with specific goal of recapitulating clinical measurements of physiologic and pathophysiologic CSF flow patterns in high-throughput bioreactor chambers. The AIMS capability to simultaneously control flow directionality, compliance, bulk flow rate, pulsation rate and peak amplitude combined with the potential to incorporate biological components provide a promising platform for investigating the direct interaction between CSF, immune cells, and shunt hardware, thereby enhancing our understanding of hydrocephalus and improve treatment strategies in the future studies.

## Electronic supplementary material

Below is the link to the electronic supplementary material.

Supplementary Material 1

Supplementary Material 2

## Data Availability

Data is provided within the manuscript or supplementary information. Further detail is available through the corresponding author.
