# Supplementary material for: Recapitulation of physiologic and pathophysiologic pulsatile CSF flow in purpose-built high-throughput hydrocephalus bioreactors
Source: Fluids Barriers CNS. 2024 Dec 19;21:103. doi: 10.1186/s12987-024-00600-1 (PMC11656981; doi:10.1186/s12987-024-00600-1)
Supplement: Supplementary file 2 — Supplementary Material 2 [file 12987_2024_600_MOESM2_ESM.docx]

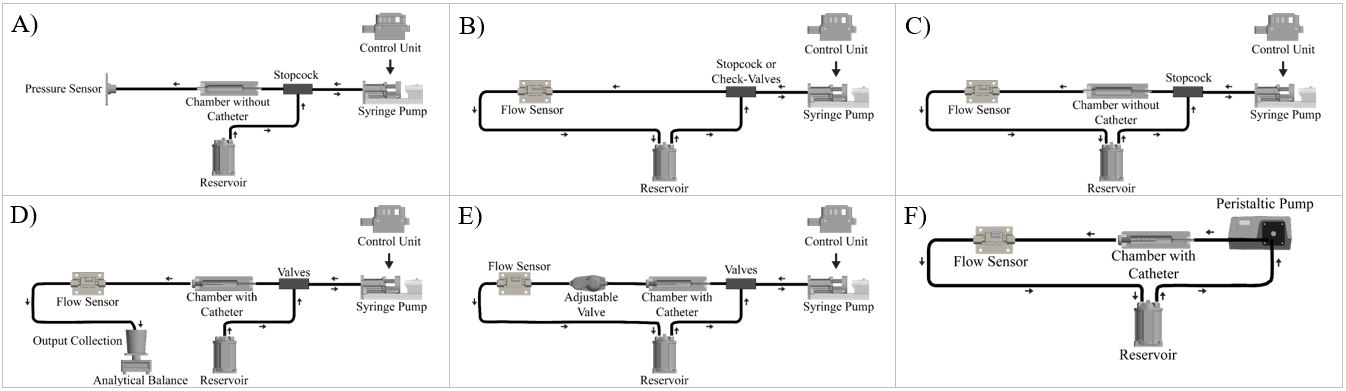


**S. Fig 1. The schematic of the fluidic circuits utilized in this study. (A)** The modified setup for the evaluation of the bioreactor chamber seal and peak pressure testing. **(B)** The setup for evaluation of Reciprocating Positive Displacement Pump peak amplitude with check valves or stopcocks **(C)** chamber consistency and peak amplitude evaluation setup **(D)** The layout of the volumetric analysis setup **(E)** the schematic of the setup with commercial adjustable valves **(F)** The setup utilized for bioreactor chamber compatibility test

| 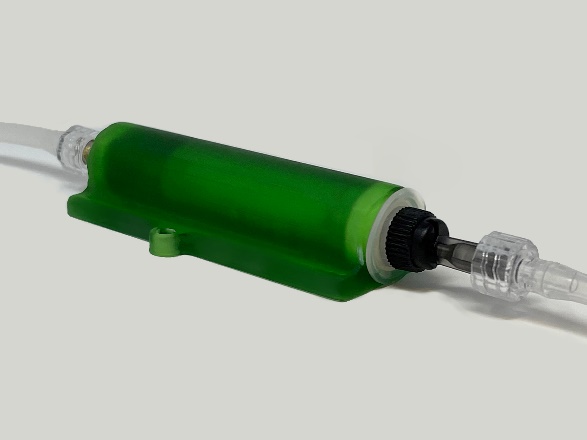  A) | 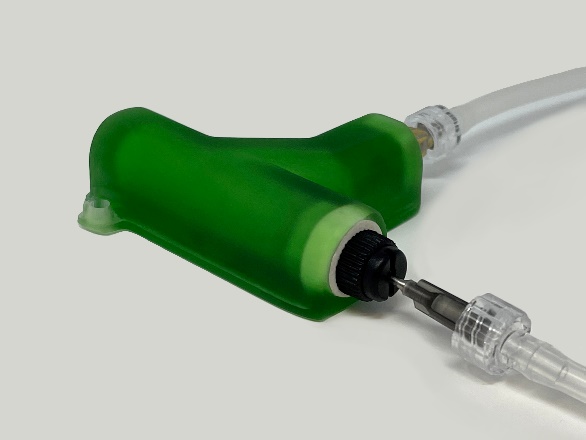  B) |
| --- | --- |
| 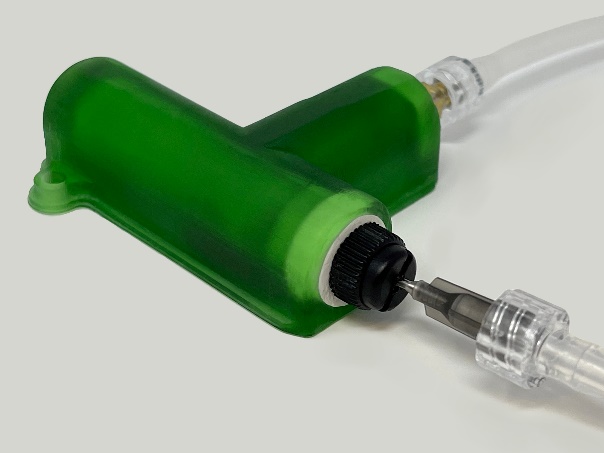  C) | 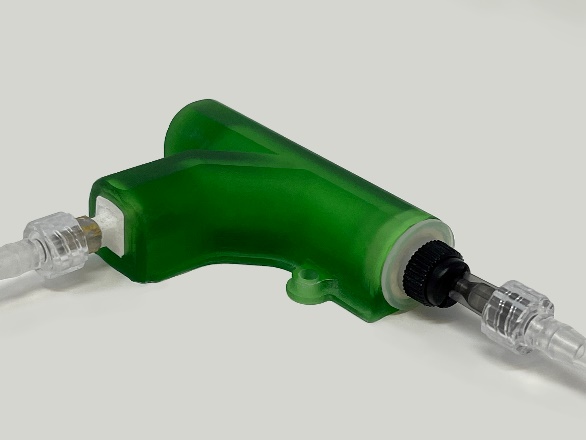  D) |

**S. Fig. 2. The four iterations of the single inlet Resin chamber. (A)** A representative of single inlet chambers with 0° inlet angle relative to the ventricular catheter. **(B)** A representative of single inlet chambers with a 45° inlet angle relative to the ventricular catheter. **(C)** A representative of single inlet chambers with a 90° inlet angle relative to the ventricular catheter. **(D)** A representative of single inlet chambers with a 135° inlet angle relative to the ventricular catheter.


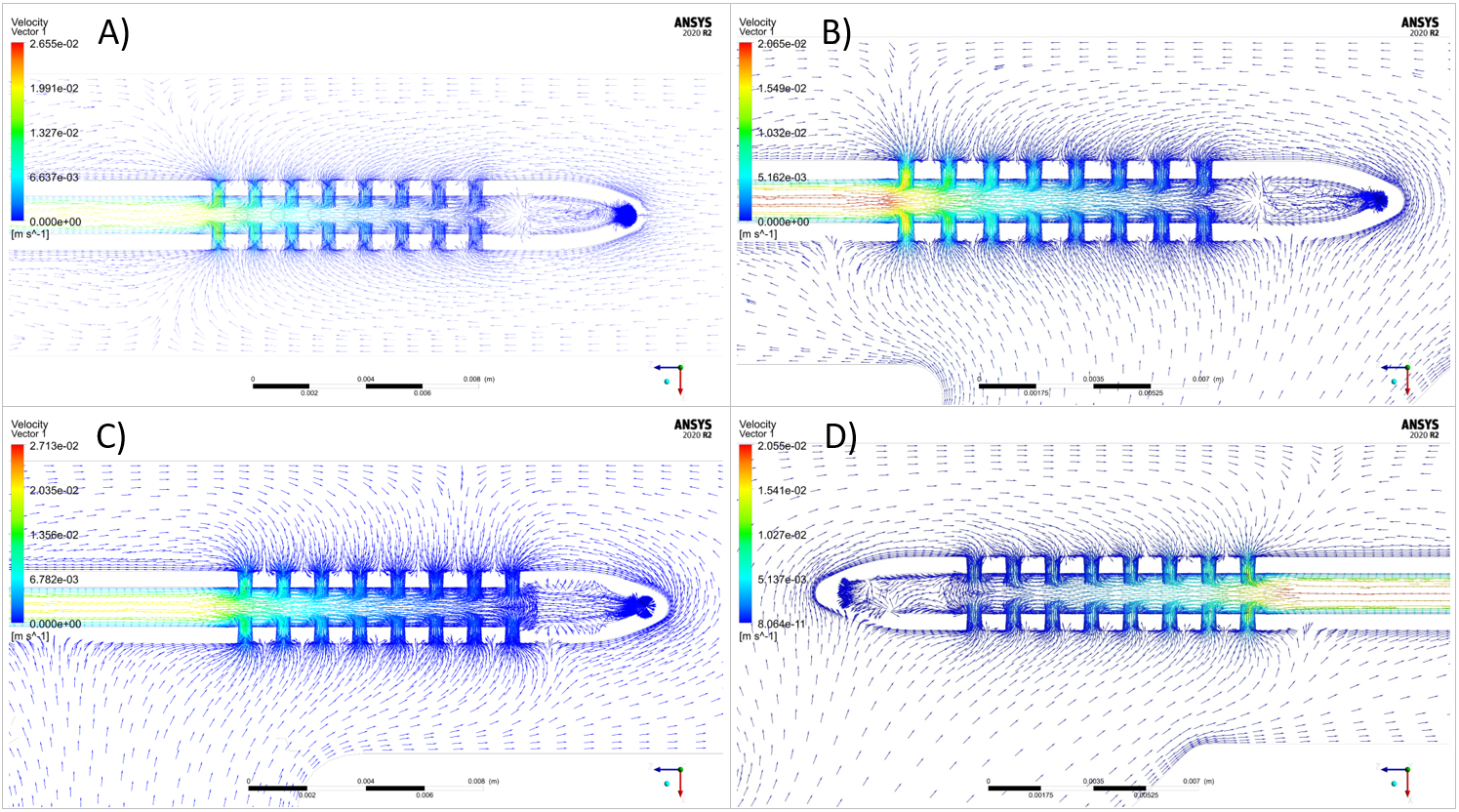


**S. Fig. 3. Visual representation of flow vector directionality in the vicinity of a commercial ventricular catheter with eight lateral holes per row and the influence of inlet offset angle in** **the four iterations of the single inlet Resin chamber. (A)** 0° inlet angle relative to the ventricular catheter. **(B)** 45° inlet angle relative to the ventricular catheter. **(C)** A representative of single inlet chambers with a 90° inlet angle relative to the ventricular catheter. **(D** 135° inlet angle relative to the ventricular catheter.


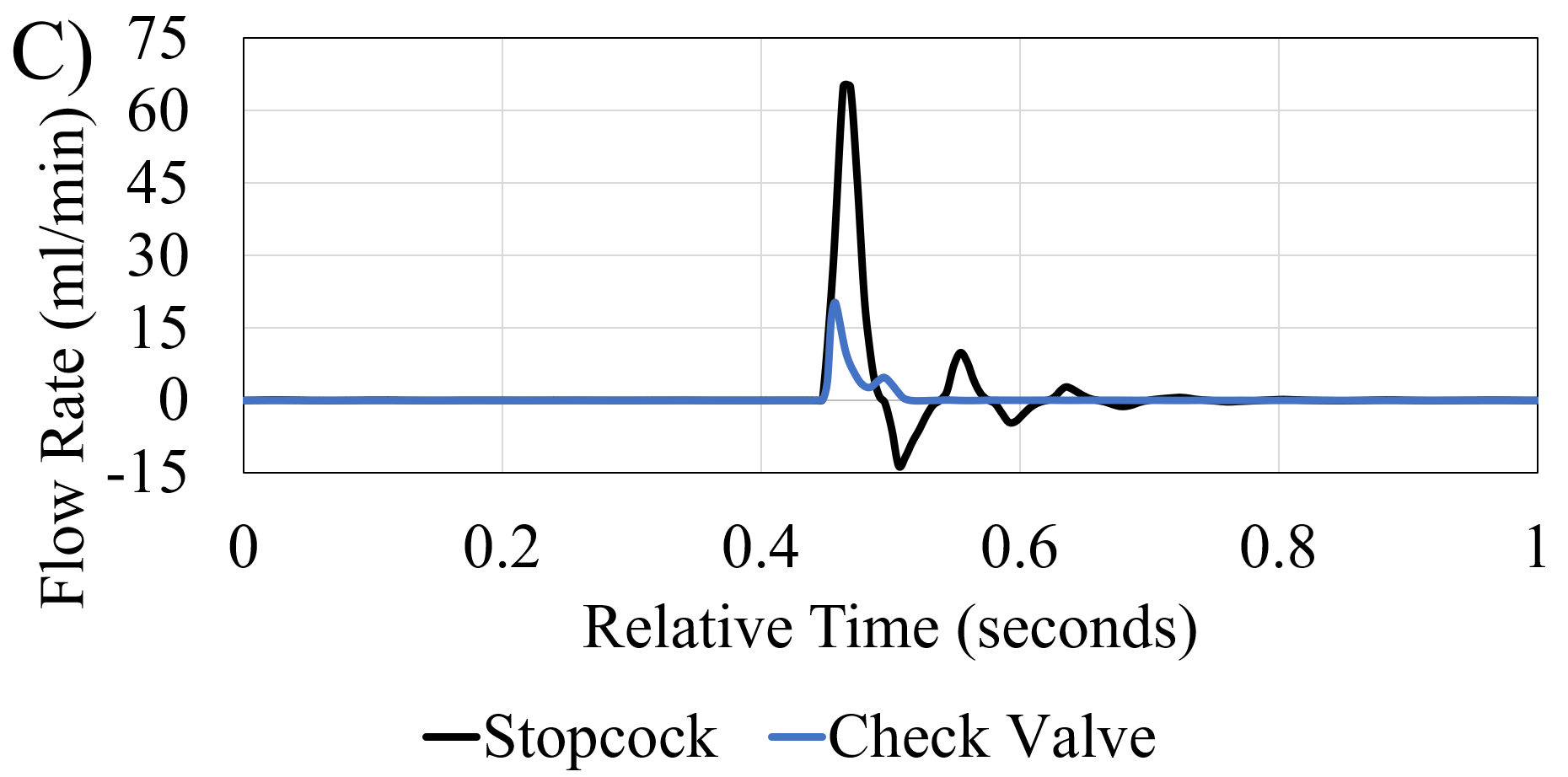


**S. Fig. 4.** Highest possible amplitude produced by the setup while maintaining a consistent bulk output rate of 0.3 ml/min bulk output rate. The setup includes the addition of a stopcock (black) and check valves (blue). The highest peak amplitude of the setup with the check valve was 27.26 ml/min while the peak amplitude of the setup with the stopcock reached 65 ml/min

**S. Table 1.** Summary of the sample number, mean, and standard deviation for the pump, single inlet resin chambers, silicone chambers, dome chambers, and PETG chambers.

| Category | Sample Number | Mean ± Standard Deviation (mL/min) |
| --- | --- | --- |
| Pump Output | 100 | 50.58 ± 4.06 |
| Resin Chambers | 400 | 30.26 ± 3.32 |
| Silicone Chambers | 400 | 50.29 ± 5.23 |
| Dome Chambers | 50 | 12.00 ± 0.47 |
| PETG Chambers | 500 | 3.77 ± 1.33 |

**S. Table 2. The impact of augmented compliance on peak amplitude and flow waveform**. Each category's sample size and mean of measured peak amplitude are indicated.

| Category | Sample Number | Mean ± Standard Deviation (ml/min) |
| --- | --- | --- |
| Pump Output- 0 µL | 10 | 5.79 ± 0.52 |
| Pump Output- 56.25 µL | 10 | 3.34 ± 0.21 |
| Pump Output-112.5 µL | 10 | 2.19 ± 0.17 |
| Pump Output- 225 µL | 10 | 0.92 ± 0.07 |
| Resin Chambers- 0 µL | 10 | 8.32 ± 0.53 |
| Resin Chambers- 56.25 µL | 10 | 4.72 ± 0.24 |
| Resin Chambers- 112.5 µL | 10 | 3.02 ± 0.27 |
| Resin Chambers- 225 µL | 10 | 1.29 ± 0.06 |
| Silicone Chambers- 0 µL | 10 | 7.27 ± 0.52 |
| Silicone Chambers- 56.25 µL | 10 | 4.60 ± 0.23 |
| Silicone Chambers- 112.5 µL | 10 | 1.74 ± 0.11 |
| Silicone Chambers- 225 µL | 10 | 1.18 ± 0.16 |
| Dome Chambers- 0 µL | 10 | 4.89 ± 0.45 |
| Dome Chambers- 56.25 µL | 10 | 1.79 ± 0.19 |
| Dome Chambers- 112.5 µL | 10 | 1.12 ± 0.1 |
| Dome Chambers- 225 µL | 10 | 0.22 ± 0.03 |
| PETG Chambers- 0 µL | 10 | 1.89 ± 0.13 |
| PETG Chambers- 56.25 µL | 10 | 1.35 ± 0.09 |
| PETG Chambers- 112.5 µL | 10 | 0.99 ± 0.09 |
| PETG Chambers- 225 µL | 10 | 0.7 ± 0.14 |

| 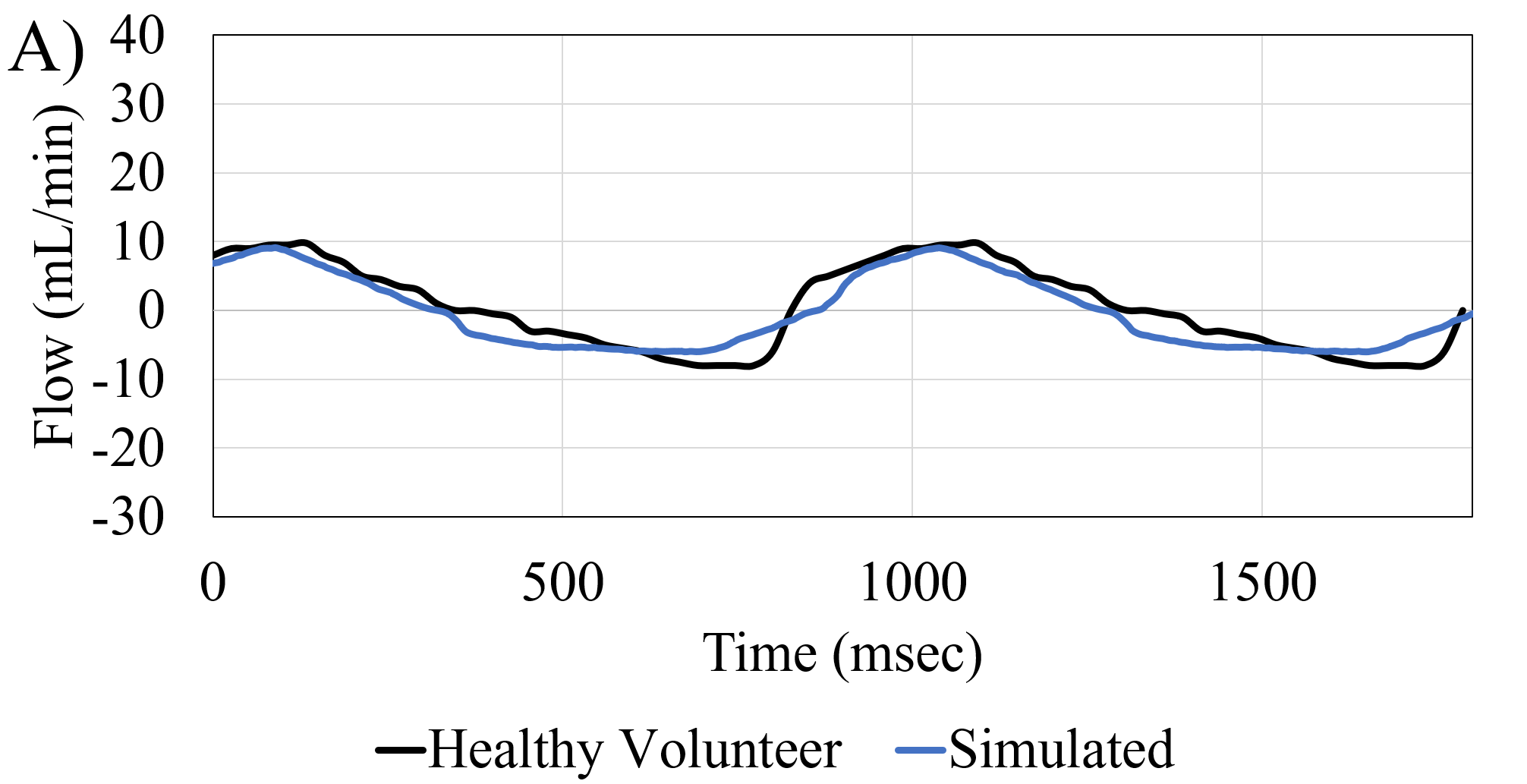 | 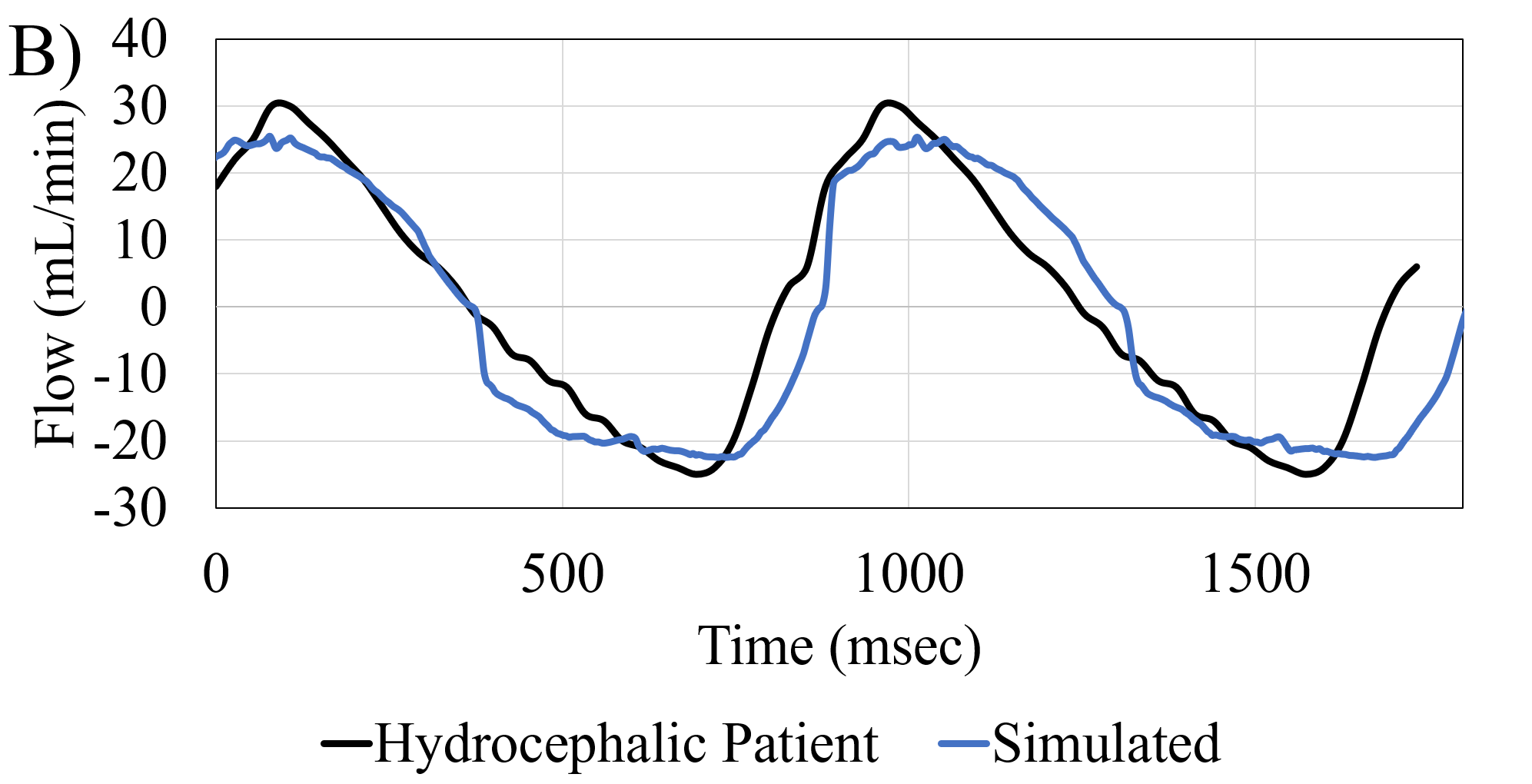 |
| --- | --- |

**S. Fig. 5 Experimental Flow Patterns** Recapitulation of Aqueduct CSF flow using the custom-profile feature of the AIMS setup in **A)** healthy volunteer, **B)** Hydrocephalic patient.

**
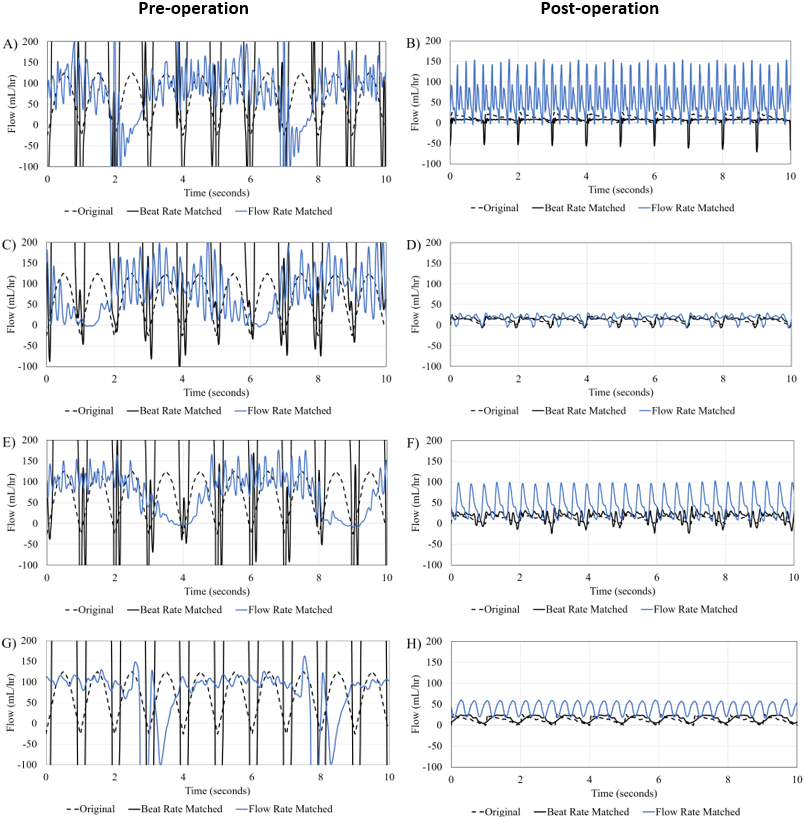
**

**S. Fig 6. Recapitulation of clinical measurements of CSF Flow through an external ventricular drain (EVD) in a pediatric patient pre-recovery and post-recovery in four-chamber types with a peristaltic pump**
